# Cataract and glaucoma surgery in microphthalmic, nanophthalmic, and high hyperopic eyes: a systematic review and meta-analysis

**DOI:** 10.3389/fopht.2026.1872292

**Published:** 2026-07-14

**Authors:** Rebecca Zheng Li, Abdullah Virk, David Bulanov, Justin Zhang, Eugene Wang, Henry Qin, Trang Bui, Karen Allison

**Affiliations:** 1 Flaum Eye Institute, University of Rochester, Rochester, NY, United States; 2 The University of Arizona College of Medicine Pheonix, Phoenix, AZ, United States; 3 University of Maryland Medical Center, Baltimore, MD, United States; 4 University System of Maryland, Baltimore, MD, United States; 5 Department of Mathematics and Statistics, University of Saskatchewan, Saskatoon, SK, Canada

**Keywords:** acute angle closure glaucoma, cataract surgery and glaucoma, glaucoma, high hyperopia, microophthalmic, nanophthalmic eyes

## Abstract

**Topic:**

This systematic review and meta-analysis evaluates refractive error (RE), visual acuity (VA), intraocular pressure (IOP), and anterior chamber depth (ACD) outcomes following surgical procedures in adults (≥ 18 years) with nanophthalmos, microphthalmos, and high hyperopia.

**Clinical relevance:**

Nanophthalmos, microphthalmos, and high hyperopia are a spectrum of anatomic variants characterized by short axial length and crowded anterior segment. These features create significant risks for angle-closure glaucoma and complex cataract surgery. As existing literature is fragmented and limited to small case series, this study provides a comprehensive evaluation of surgical safety and efficacy in these high-risk populations.

**Methods:**

Following Preferred Reporting Items for Systematic Reviews and Meta-Analyses (PRISMA) guidelines, a search was conducted across five databases (Cochrane, PubMed, Scopus, Ovid MEDLINE, and Embase via Ovid) for studies published between January 2010 and December 2024. Eligible studies reported original clinical data on surgical interventions for adult (≥ 18 years) patients with nanophthalmos, microphthalmos, or high hyperopia with concomitant cataract or glaucoma. Meta-analysis, subgroup analysis, and meta-regression were performed to assess changes in RE, VA, IOP, and ACD.

**Results:**

Meta-analysis of 21 articles involving 957 patients (1,110 eyes) suggests that surgical intervention is associated with improvement in clinical parameters across a spectrum of short eyes. Standalone phacoemulsification significantly reduced RE in nanophthalmic eyes (mean difference (MD) = -13.20 diopters (D); 95% confidence interval (CI): -15.19, -11.22; Q = 3.51, p = 0.48, I^2^ = 0.0%) at medium-term follow-up and in high hyperopic eyes (MD = -9.23 D; 95% CI: -15.02, -3.45; Q = 322.19, p < 0.001, I^2^ = 97.8%) at medium-term follow-up with high heterogeneity in the effects. Standalone phacoemulsification significantly improved VA in nanophthalmic eyes at both short-term (MD = -0.66 logarithm of the minimum angle of resolution (logMAR); 95% CI: -1.30, -0.02; Q = 98.45, p < 0.001, I^2^ = 97.0%) and medium-term (MD = -0.70 logMAR; 95% CI: -1.30, -0.09; Q = 97.14, p < 0.001, I^2^ = 93.8%) follow-up with high heterogeneity in the effects. In contrast, phacoemulsification + filtering surgery resulted in a significant worsening in VA at short-term follow-up for nanophthalmic eyes (MD = 0.28 logMAR; 95% CI: 0.21, 0.35; Q = 1.24, p = 0.27, I^2^ = 19.4%). Significant IOP reduction was achieved primarily through combined procedures in both nanophthalmic and microphthalmic eyes. Presence of concomitant glaucoma was associated with a greater reduction in IOP across all axial lengths. Significant ACD deepening was achieved across all axial lengths (MD = 1.30 millimeters (mm), 95% CI: 0.86, 1.74; Q = 244.55, p < 0.001, I^2^ = 99.3%) with high heterogeneity in the effects. Meta-regression identified a significant study-level association between mean age and post-operative RE (multiple adjusted coefficient: 10.72, p = 0.001). No effect of race was detectable in this study-level meta-regression, although power was limited.

**Conclusion:**

Surgical interventions in adults with nanophthalmos, microphthalmos, and high hyperopia were associated with meaningful improvements in RE, VA, IOP, and ACD, although outcomes varied by underlying diagnosis and procedure type. Standalone phacoemulsification consistently improved refractive and anatomical outcomes, particularly in nanophthalmos and high hyperopia, while combined procedures incorporating vitrectomy or filtering surgery achieved greater IOP reduction in eyes with concomitant glaucoma. Subgroup analyses suggested that shorter axial length and the presence of glaucoma influenced treatment response. However, substantial heterogeneity across studies and frequent post-operative complications highlight the need for standardized surgical protocols and higher-quality prospective studies to better define optimal management strategies for these anatomically complex eyes.

**Systematic Review Registration:**

https://www.crd.york.ac.uk/PROSPERO/view/CRD420251033701, identifier CRD420251033701.

## Introduction

Microphthalmos, nanophthalmos, and high hyperopia represent a spectrum of anatomic variants characterized by reduced globe size, crowded anterior segment, and increased susceptibility to angle closure disease (1–6). Microphthalmos is defined as small eyes with structural abnormalities, such as colobomas, and axial length (AL) < 21 mm (7–9). Nanophthalmos is characterized by small but otherwise structurally normal eyes (1, 10, 11). The key defining features of nanophthalmos include AL < 20 mm (4, 11, 12), shallow anterior chamber < 2.5 mm (12, 13), and thickened sclera (14, 15). High hyperopia, often defined as greater than +8 diopters (D), frequently coexists with these anatomic findings and contributes to surgical challenges (1, 6, 14). These eyes are predisposed to angle closure glaucoma (14), uveal effusion syndrome (16, 17), and exudative retinal detachment (18) due to their configuration, and cataract surgery, glaucoma surgery, and combined procedures are all required. Yet, surgical management remains complex with inconsistent outcomes reported across small case series.

Cataract surgery, the most frequently performed ophthalmic procedure worldwide, is significantly more complex in eyes with microphthalmos, nanophthalmos, or high hyperopia (19–22). The combination of small anterior segment dimensions, thickened sclera, and increased vitreous pressure substantially elevates the risk of a broad spectrum of intraoperative and post-operative complications (20, 23, 24). Documented complications include uveal effusion, cystoid macular edema, corneal decompensation, malignant glaucoma, vitreous loss, suprachoroidal hemorrhage, and retinal detachment (11, 21, 25–27). Notably, a large case series reported that eyes with an AL shorter than 20 mm have a 15- to 20-fold higher risk of surgical complications compared to eyes with average axial length (28). Although strategies such as pre-operative administration of hyperosmotics or corticosteroids, prophylactic sclerostomy, and optimized phacoemulsification settings may reduce risk, complication rates remain substantially higher than in the general population (20, 22, 28).

Glaucoma management in patients with microphthalmos, nanophthalmos, or high hyperopia is similarly challenging. Medical therapy is frequently inadequate, and conventional surgical interventions each present distinct risks in these eyes (14, 27, 29). For instance, laser peripheral iridotomy may not prevent progressive synechial angle closure in eyes with persistently crowded anterior segments (30). Filtration procedures such as trabeculectomy are associated with a disproportionately high incidence of choroidal effusion, uveal effusion syndrome, and malignant glaucoma in this population (29). Tube shunts and minimally invasive glaucoma surgeries (MIGS) have been utilized in select cases, but available data are limited to small series or isolated reports (31, 32). Even lens extraction, which is increasingly recognized as a primary treatment for angle-closure glaucoma in the general population, remains technically demanding and high-risk in microphthalmic, nanophthalmic, or hyperopic eyes (11, 27, 33). These considerations require clinicians to carefully balance the urgent need for intraocular pressure (IOP) control or visual rehabilitation against significant operative risks.

Despite the significance of this issue, most studies examining cataract or glaucoma surgery in microphthalmic, nanophthalmic, or high hyperopic eyes are limited to small case reports or series, which restricts the generalizability of their findings (14, 21, 25, 29, 34, 35). Although a protocol has been published for a systematic review of complications in nanophthalmos and microphthalmos (35), there is no completed synthesis focused on specifically addressing treatment outcomes, surgical decision-making, or the role of cataract extraction in conjunction with glaucoma management. A comprehensive synthesis of outcomes across a spectrum of short eyes is therefore needed to guide clinical decision making.

This systematic review aims to evaluate the effectiveness and safety of cataract surgery and glaucoma procedures in adults (≥ 18 years) with microphthalmos, nanophthalmos, or high hyperopia with narrow angles or concomitant angle-closure glaucoma. Additional objectives include identifying gaps in the literature and summarizing the current consensus regarding optimal treatment strategies.

This systematic review and meta-analysis addresses three primary research questions:

1. How effective is surgical intervention in correcting anatomical and physiological ocular parameters in adult (≥ 18 years) patients with microphthalmos, nanophthalmos, or high hyperopia?
2. What surgical technique(s) yield better clinical outcomes for adult (≥ 18 years) patients with microphthalmos, nanophthalmos, or high hyperopia?
3. How do ocular disease severity and demographic factors influence surgical success?

Through systematic synthesis of the available evidence, this study aims to inform clinical decision-making, highlight strategies to reduce surgical risk, and identify priorities for future research.

## Methods

This systematic review and meta-analysis was conducted in accordance with the Preferred Reporting Items for Systematic Reviews and Meta-Analyses (PRISMA) 2020 statement and the Guidelines for Reporting Reliability and Agreement Studies (36, 37). The study protocol was registered with PROSPERO (Registration ID: CRD420251033701). This study did not meet the criteria for human subjects research as defined by the University of Rochester Institutional Review Board as it did not include patient data. Therefore, it did not require institutional review board approval or informed consent. This study adhered to the Declaration of Helsinki.

### Search strategy

A thorough literature search was conducted on electronic databases including Cochrane Library, PubMed, Scopus, Ovid MEDLINE, and Embase via Ovid for articles published between January 2010 and December 2024. The search strategy was developed in conjunction with the content expert (KA). Database searches involved keywords and Medical Subject Headings (MeSH) terms related to nanophthalmos, microphthalmos, high hyperopia, angle-closure, and narrow angles, combined with relevant treatment terms, such as laser peripheral iridotomy (LPI), laser goniotomy, and cataract surgery. The full search strategy used is given in **Supplementary Table 1**.

### Eligibility criteria and study selection

Abstracts queried from research databases following the search terms in **Supplementary Table 1** were collected and screened for relevance. For title and abstract screening, all articles were independently reviewed by two authors (RL, AV, DB, EW, and HQ), and discrepancies were addressed by a third reviewer. Duplicates were screened out and excluded. Full-text articles were acquired for abstracts that progressed to full-text screening.

Full-text screening was conducted by having two reviewers independently screen each article for eligibility (RL, AV, DB, EW, and HQ), and discrepancies were addressed by a third reviewer. Published full-texts were included based on the following criteria: (1) The study is an English-language study containing original clinical data published between January 2010 and December 2024; (2) the primary condition of interest in the article is nanophthalmos, microphthalmos, or high hyperopia; (3) these patients also have either cataracts, angle-closure glaucoma, or narrow angle glaucoma; (4) involve a procedure to address the cataract or glaucoma pathology, such as cataract surgery or LPI; (5) outcome measures for these procedures such as refractive error (RE) in diopters (D), visual acuity (VA) in logarithm of the minimum angle of resolution (logMAR), intraocular pressure (IOP) in millimeters of mercury (mmHg), or anterior chamber depth (ACD) in millimeters (mm) were included. Specifically, studies reporting VA in Snellen or decimal notation were converted to logMAR units using standard published conversion formulas to enable quantitative synthesis across studies. Snellen acuity values were first converted to decimal acuity and then transformed to logMAR using the formula: logMAR = -log_10_(decimal acuity), where higher logMAR values indicate worse VA. The January 2010 search cutoff was selected to emphasize contemporary surgical techniques, modern intraocular lens and glaucoma device technologies, and current perioperative management practices relevant to present-day clinical care. Earlier landmark studies were reviewed for historical context and discussion. Studies were excluded if they focused on pediatric populations (< 18 years), pseudophakic patients, or patients with multiple additional ophthalmic comorbidities (e.g. amblyopia or strabismus). Specifically, studies involving eyes that were pseudophakic prior to the intervention were excluded; however, studies evaluating piggyback intraocular lens (IOL) implantation as part of the surgical intervention strategy were eligible for inclusion. Animal studies, reviews, conference abstracts, guidelines, editorials, commentaries, and opinion pieces were also excluded.

### Data extraction and quality assessment

Data from each full-text article was then extracted to a pre-made Google Sheets template independently by two authors (RL, DB, EW, and JZ), with discrepancies addressed by a third reviewer. A standardized format was used to extract the following information: (1) study information [study name, first author, year of publication, study location, study design]; (2) basic study data [inclusion criteria, exclusion criteria, total patient count, male patient count, female patient count, mean age, intervention(s)]; (3) outcome measures [pre- and post-op RE, VA, IOP, ACD, and post-op ophthalmic complications].

Evidence quality assessment of eligible studies was conducted using the Risk Of Bias In Non-randomized Studies – of Interventions, Version 2 (ROBINS-I V2) for non-randomized studies and the risk of bias in randomized trials (ROB-2) for randomized studies (38, 39). Three authors (RL, DB, and JZ) independently evaluated each article to determine the risk of bias for each article; in cases of disagreement, a third reviewer was brought in to help review and reach a consensus.

### Statistical analysis

Meta-analysis was conducted using R to evaluate the efficacy of various surgical interventions on clinical outcomes in adult (≥ 18 years) patients with nanophthalmos, microphthalmos, or high hyperopia. Surgical interventions were categorized *a priori* according to clinically distinct operative approaches with differing mechanisms of intraocular pressure reduction and anterior segment modification, including standalone phacoemulsification, phacoemulsification + goniosynechialysis, phacoemulsification + vitrectomy, phacoemulsification + filtering surgery, and standalone laser peripheral iridotomy/trabeculectomy/tube shunt procedures. The primary clinical outcomes analyzed included RE, VA, IOP, and ACD. Follow-up timepoints were stratified *a priori* into short term (≤ 3 months), medium term (4 to 12 months), and long term (> 12 months) intervals to evaluate immediate postoperative effects, intermediate recovery and stabilization, and durability of surgical outcomes over time. The above data analysis stratification was done to systematically investigate potential sources of heterogeneity.

Pooled effect sizes were calculated using the pre- and post-operative mean difference (MD), which yielded pooled MD estimates and the corresponding 95% confidence intervals (CI). MD rather than standardized mean difference was used, because although measurement techniques may have varied between studies, the reported outcome metrics remained directly comparable on identical continuous scales. Statistical significance for these pooled results was determined based on whether the 95% CI crossed the null value (MD = 0). Due to the heterogeneity in the population and methodologies among the studies, we default to use the random effects model following the guidance from Harrer et al. (40) Cochran’s Q and I^2^ were calculated to detect between-study heterogeneity and reported for all pooled estimates. To evaluate the robustness of the pooled estimates and assess the influence of individual studies, leave-one-out sensitivity analysis was performed for analyses with high heterogeneity (I^2^ > 50%).

Subgroup analysis was performed to assess post-operative ACD, IOP, RE, and VA outcomes based on disease severity according to axial length (AL) (AL < 18 mm, AL 18–20 mm, and AL 20–22 mm; with or without concomitant glaucoma). These stratifications were selected based on the biologic rationale that increasing axial shortening and coexisting glaucoma are associated with greater anterior segment crowding, altered ocular anatomy, and higher surgical complexity, which may influence both efficacy and complication risk. The AL-based strata were not intended to replicate the diagnostic categories of microphthalmos, nanophthalmos, and high hyperopia, which additionally incorporate structural and refractive characteristics. Rather, the AL 20–22 mm subgroup primarily represented highly hyperopic eyes with crowded anterior segment anatomy but less severe axial shortening.

To identify the effect of age and race on surgical outcomes, a meta-regression was performed.

Meta-regression adjusted coefficients and p-values helped determine the association of these two demographic factors on the magnitude of change in RE, VA, IOP, and ACD. A p-value of <0.05 was considered statistically significant for these associations. Race was included as a meta-regression covariate based on the Primary Angle-Closure Disease Preferred Practice Pattern (11) statement that Asian descent is a risk factor for primary angle closure disease. Race was categorized as Asian or non-Asian based on the race of the majority population in the country where each included study took place.

Post-operative ophthalmic complications were excluded from quantitative analysis and summarized narratively since all studies reported complications using qualitative descriptions instead of quantitative data.

Publication bias was assessed using Egger’s regression test and visual inspection of funnel plots for forest plots including at least 10 studies, consistent with recommendations that these methods are unreliable with smaller number of studies. For forest plots with fewer than 10 studies, publication bias was not formally assessed.

## Results

### Study selection

**Figure 1:**
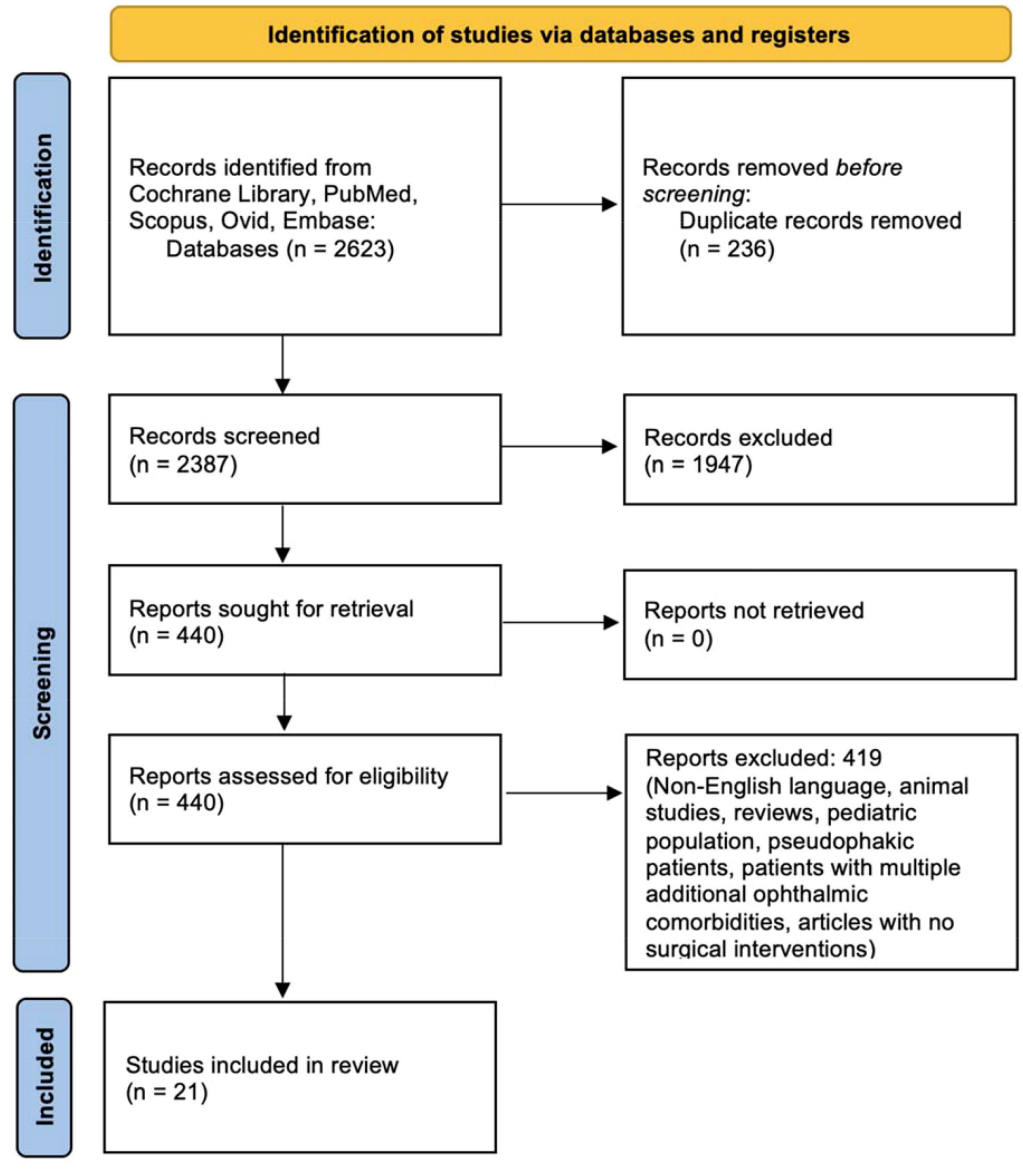
PRISMA flowchart.

A study flow diagram, which outlines the results of our search strategy, is presented in **Figure 1**. An electronic search using Cochrane Library, PubMed, Scopus, Ovid, and Embase queried 2623 articles, of which 236 duplicates were removed. The remaining 2387 articles were screened by title and abstract and resulted in 440 articles for full-text review. After the inclusion/exclusion criteria were implemented, a total of 21 articles involving 957 patients (1,110 eyes) were included in the review and meta-analysis. The characteristics and results of the 21 included studies are summarized in **Supplementary Table 2**.

### Risk of bias assessment

**Figure 2:**
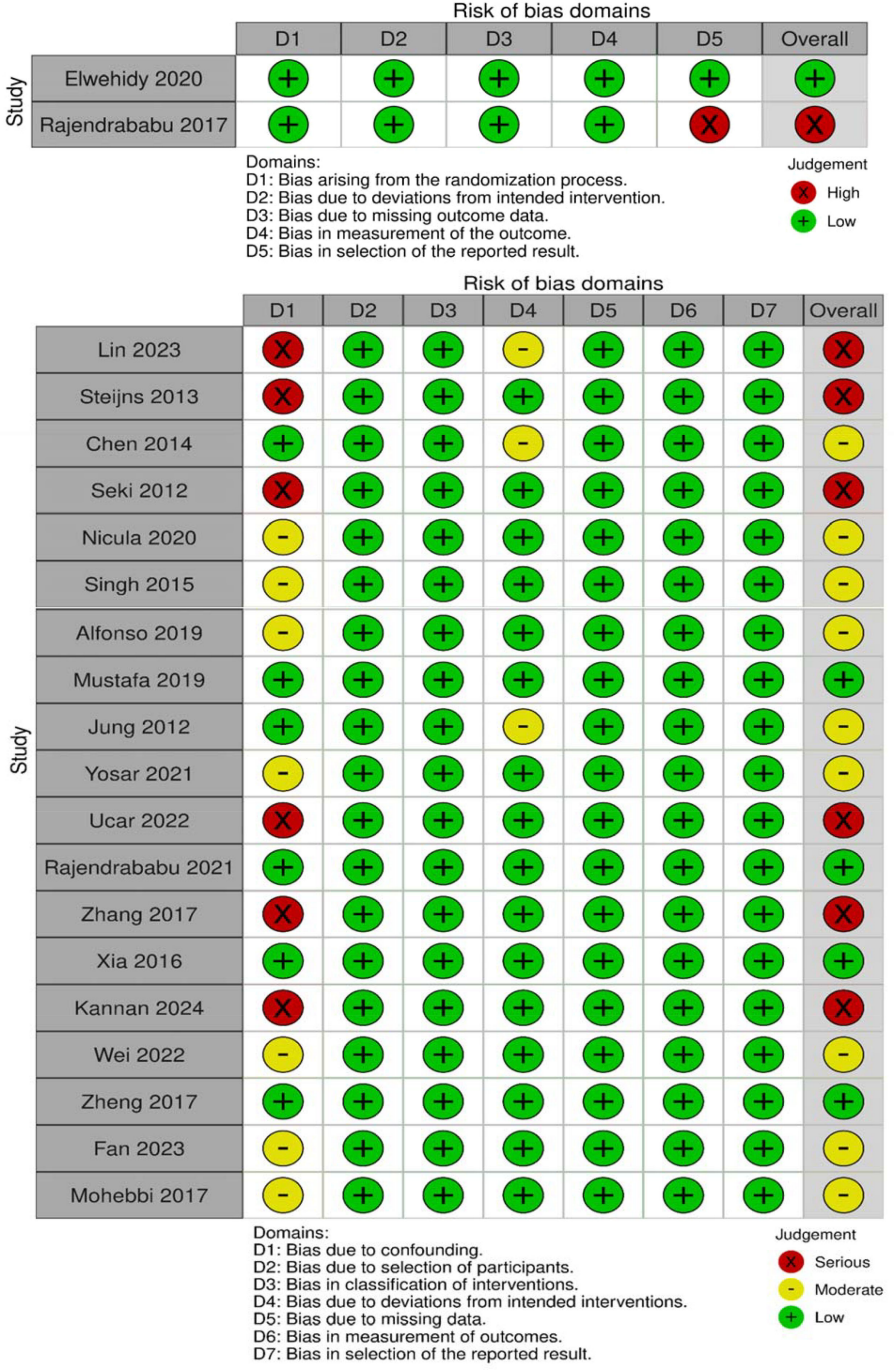
Traffic light plots of the risk-of-bias assessment results of randomized studies with ROB-2 (top) and of non-randomized studies with ROBINS-I version 2 (bottom).

Risk-of-bias assessment for randomized and non-randomized studies is presented in **Figure 2**. Two studies were randomized and 19 were non-randomized. Using the Cochrane risk-of-bias tool for randomized trials (ROB-2), one study was considered to have an overall low risk of bias, while another study was considered to have an overall high risk of bias due to bias in selection of the reported result. Using the Cochrane Risk of Bias in Non-Randomized Studies – of Interventions (ROBINS-I) tool, the overall risk of bias for non-randomized studies was moderate. The domain with the highest overall level of risk was the domain for bias due to confounding.

### Results of syntheses

Meta-analysis was performed to evaluate the efficacy of standalone phacoemulsification, phacoemulsification + goniosynechialysis, phacoemulsification + vitrectomy, phacoemulsification + filtering surgery, and standalone procedure laser peripheral iridotomy/trabeculectomy/tube shunt procedures on RE, VA, IOP, and ACD in adult (≥ 18 years) patients with nanophthalmos, microphthalmos, and high hyperopia across short (≤ 3 months), medium (4 to 12 months), and long (> 12 months) term follow-up timepoints.

#### Refractive outcomes

**Figure 3:**
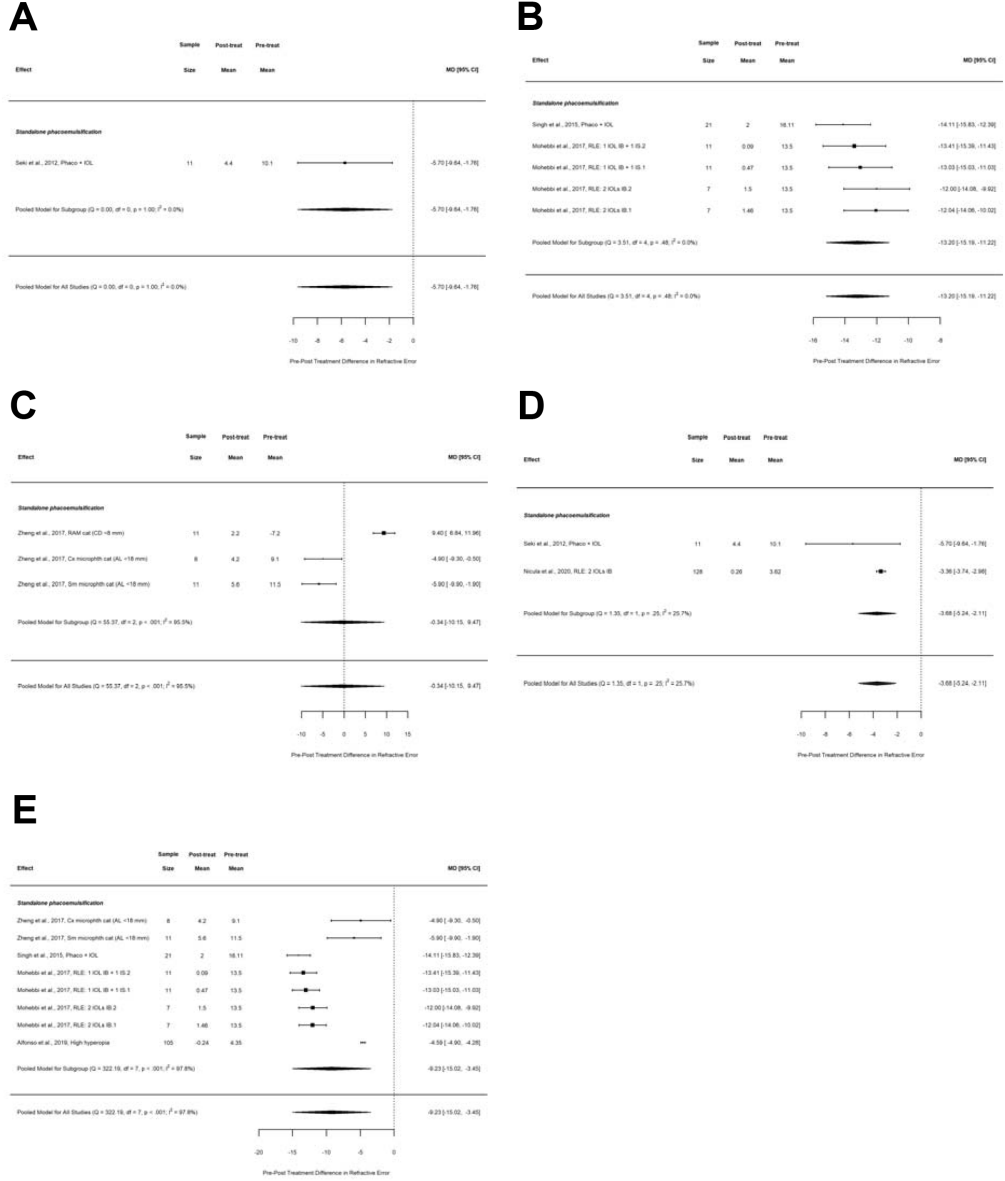
Forest plots showing the mean difference in refractive error pre- and post-treatment for patients with **(A)** nanophthalmos at short-term follow-up, **(B)** nanophthalmos at medium-term follow-up, **(C)** microphthalmos at medium-term follow-up, **(D)** high hyperopia at short-term follow-up, and **(E)** high hyperopia at medium-term follow-up, subgrouped by surgical technique.

Standalone phacoemulsification resulted in a significant reduction in RE for patients with nanophthalmos across both short- and medium-term follow-up periods. In the short-term, a single study reported a significant decrease in RE with a pre- and post-operative MD of -5.70 D (95% CI: -9.64, -1.76) (**Figure 3A**). Because this estimate is derived from one study, it should be interpreted cautiously and considered preliminary. In the medium-term, the pooled MD for five studies demonstrated a significant reduction in refractive error of -13.20 D (95% CI: -15.19, -11.22; Q = 3.51, p = 0.48, I^2^ = 0.0%) (**Figure 3B**).

In contrast to the nanophthalmic cohort, standalone phacoemulsification resulted in a non-significant overall change in RE for patients with microphthalmos at the medium-term follow-up period (MD = -0.34 D; 95% CI: -10.15, 9.47; Q = 55.37, p < 0.001, I^2^ = 95.5%) (**Figure 3C**) with high heterogeneity in the effects. Notably, patients with relative anterior microphthalmos (RAM) cataracts and small corneal diameters (CD) (CD < 8mm) experienced a change in the direction of increased RE, while patients with complex and simple microphthalmos experienced a change in the opposite direction, suggesting more evidence is needed.

Lastly, standalone phacoemulsification resulted in a significant reduction in RE for patients with high hyperopia across both short- and medium-term follow-up periods. In the short-term, the pooled MD for two studies demonstrated a significant decrease in RE of -3.68 D (95% CI: -5.24, -2.11; Q = 1.35, p = 0.25, I^2^ = 25.7%) (**Figure 3D**). In the medium-term, the pooled MD for eight studies demonstrated a significant reduction in refractive error of -9.23 D (95% CI: -15.02, -3.45; Q = 322.19, p < 0.001, I^2^ = 97.8%) (**Figure 3E**) with high heterogeneity in the effects. Notably, all eight studies report effects in the same direction, suggesting the direction of the overall effect.

Leave-one-out sensitivity analyses demonstrated that no individual study substantially altered the magnitude, direction, or statistical significance of the pooled estimates (**Supplementary Figure 1**).

#### Visual acuity outcomes

**Figure 4:**
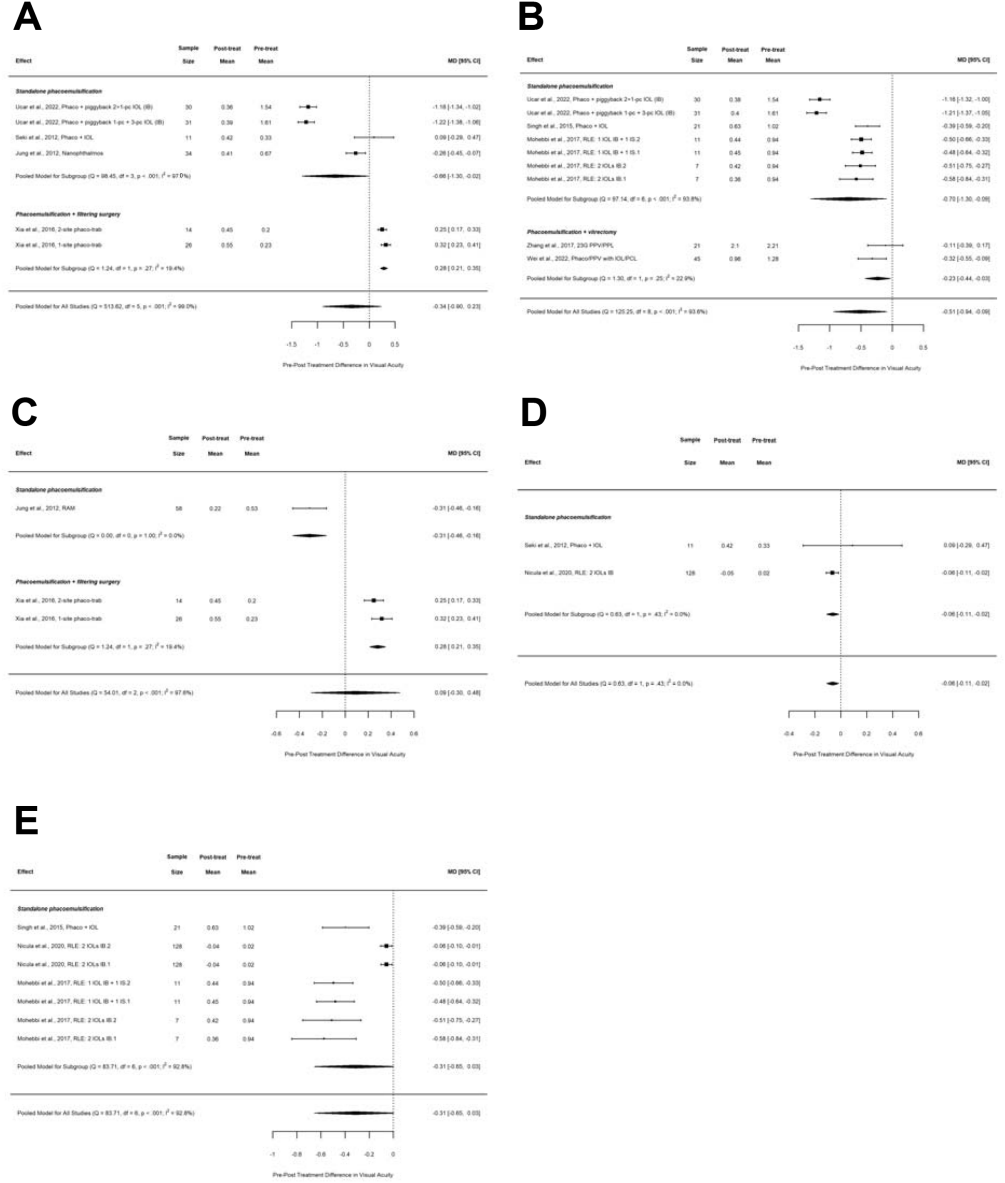
Forest plot showing the mean difference in visual acuity pre- and post-treatment for patients with **(A)** nanophthalmos at short-term follow-up, **(B)** nanophthalmos at medium-term follow-up, **(C)** microphthalmos at short-term follow-up, **(D)** high hyperopia at short-term follow-up, and **(E)** high hyperopia at medium-term follow-up, subgrouped by surgical technique.

Standalone phacoemulsification resulted in a significant improvement in VA for patients with nanophthalmos at the short-term follow-up period (MD = -0.66 logMAR; 95% CI: -1.30, -0.02; Q = 98.45, p < 0.001, I^2^ = 97.0%) (**Figure 4A**) with high heterogeneity in the effects. In contrast, phacoemulsification + filtering surgery resulted in a significant short-term worsening of VA for patients with nanophthalmos, with a pooled MD of 0.28 logMAR (95% CI: 0.21, 0.35; Q = 1.24, p = 0.27, I^2^ = 19.4%) (**Figure 4A**). In the medium-term, standalone phacoemulsification for patients with nanophthalmos resulted in a significant improvement in VA (MD = -0.70 logMAR; 95% CI: -1.30, -0.09; Q = 97.14, p < 0.001, I^2^ = 93.8%) (**Figure 4B**) with high heterogeneity. Notably, all seven studies report effects in the same direction, suggesting the direction of the overall effect. Phacoemulsification + vitrectomy also resulted in a significant improvement in VA (MD = -0.23 logMAR, 95% CI: -0.44, -0.03; Q = 1.30, p = 0.25, I^2^ = 22.9%) (**Figure 4B**).

For patients with microphthalmos, a single study demonstrated that standalone phacoemulsification resulted in a significant improvement in VA in the short-term (MD = -0.31 logMAR; 95% CI: -0.46, -0.16) (**Figure 4C**). Because this estimate is derived from one study, it should be interpreted cautiously and considered preliminary. In contrast, phacoemulsification + filtering surgery resulted in a significant worsening in VA in the short-term for patients with microphthalmos (MD = 0.28 logMAR, 95% CI: 0.21, 0.35; Q = 1.24, p = 0.27, I^2^ = 19.4%) (**Figure 4C**).

Lastly, standalone phacoemulsification resulted in a significant improvement in VA for patients with high hyperopia in the short term (MD = -0.06 logMAR, 95% CI: -0.11, -0.02; Q = 0.63, p = 0.43, I^2^ = 0%) (**Figure 4D**) but a non-significant improvement in VA for patients with high hyperopia in the medium term (MD = -0.31 logMAR, 95% CI: -0.65, 0.03; Q = 83.71, p < 0.001, I^2^ = 92.8%) (**Figure 4E**) with high heterogeneity in the effects. Notably, all seven studies report effects in the same direction, suggesting the direction of the overall effect.

Leave-one-out sensitivity analyses demonstrated that no individual study substantially altered the magnitude, direction, or statistical significance of the pooled estimates (**Supplementary Figure 2**).

#### Intraocular pressure outcomes

**Figure 5:**
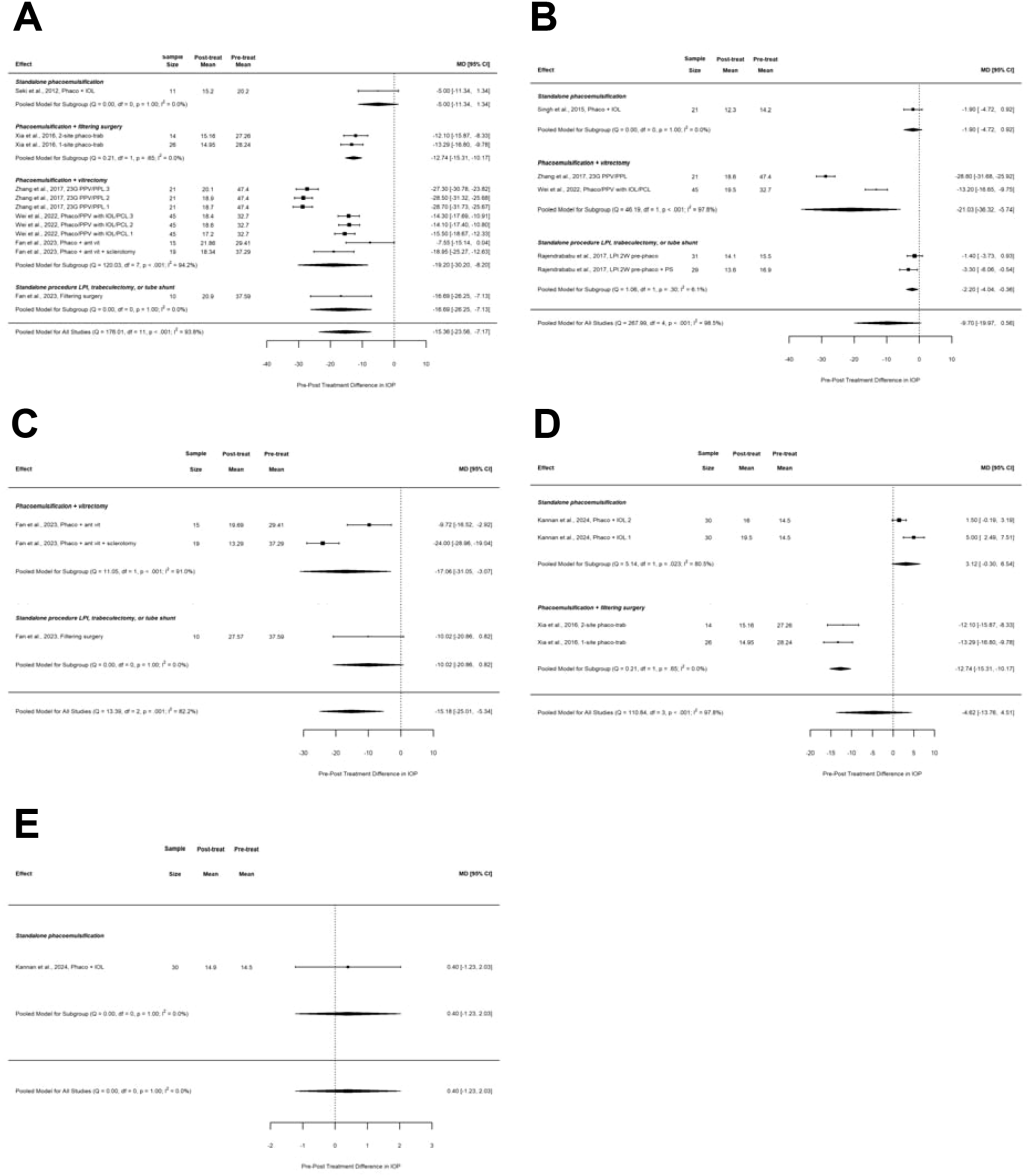
Forest plot showing the mean difference in intraocular pressure pre- and post-treatment for patients with **(A)** nanophthalmos at short-term follow-up, **(B)** nanophthalmos at medium-term follow-up, **(C)** nanophthalmos at long-term follow-up, **(D)** microphthalmos at short-term follow-up, and **(E)** microphthalmos at medium-term follow-up, subgrouped by surgical technique.

For patients with nanophthalmos, phacoemulsification + filtering surgery (MD = -12.74 mmHg; 95% CI: -15.31, -10.17; Q = 0.21, p = 0.65, I^2^ = 0%) (**Figure 5A**), phacoemulsification + vitrectomy (MD = -19.20 mmHg; 95% CI: -30.20, -8.20; Q = 120.03, p < 0.001, I^2^ = 94.2%) (**Figure 5A**), and a single study involving standalone filtering surgery (MD = -16.69 mmHg; 95% CI: -26.25, -7.13) (**Figure 5A**) resulted in a significant reduction in IOP in the short term, while a single study involving standalone phacoemulsification resulted in a non-significant reduction in IOP in the short term (MD = -5.00 mmHg; 95% CI: -11.34, 1.34) (**Figure 5A**). Because these latter two estimates are both derived from single studies, they should be interpreted cautiously and considered preliminary. Notably, there is high heterogeneity in the phacoemulsification + vitrectomy group data, though all eight studies in this group report effects in the same direction, suggesting the direction of the overall effect. Visual inspection of the funnel plot demonstrated mild asymmetry; however, Egger’s regression test was not statistically significant (p = 0.16), suggesting no clear evidence of publication bias (**Supplementary Figure 3**). In the medium term, phacoemulsification + vitrectomy (MD = -21.03 mmHg; 95% CI: -36.32, -5.74; Q = 46.19, p < 0.001, I^2^ = 97.8%) (**Figure 5B**) and standalone LPI (MD = -2.20 mmHg; 95% CI: -4.04, -0.36; Q = 1.06, p = 0.30, I^2^ = 6.1%) (**Figure 5B**) resulted in significant reductions in IOP, while a single study involving standalone phacoemulsification (MD = -1.90 mmHg; 95% CI: -4.72, 0.92) (**Figure 5B**) resulted in a non-significant IOP reduction. Because this estimate is derived from one study, it should be interpreted cautiously and considered preliminary. Notably, there is high heterogeneity in the phacoemulsification + vitrectomy group data, though both studies in this group report effects in the same direction, suggesting the direction of the overall effect. In the long term, phacoemulsification + vitrectomy resulted in a significant IOP reduction (MD = -17.06 mmHg; 95% CI: -31.05, -3.07; Q = 11.05, p < 0.001, I^2^ = 91.0%) (**Figure 5C**) with high heterogeneity in the effects. Notably, both studies in this group report effects in the same direction, suggesting the direction of the overall effect. In contrast, a single study involving standalone filtering surgery resulted in a non-significant IOP reduction (MD = -10.02 mmHg; 95% CI: -20.86, 0.82) (**Figure 5C**). Because this estimate is derived from one study, it should be interpreted cautiously and considered preliminary.

For patients with microphthalmos, standalone phacoemulsification resulted in a non-significant increase in IOP in the short term (MD = 3.12 mmHg; 95% CI: -0.30, 6.54; Q = 5.14, p = 0.023, I^2^ = 80.5%) (**Figure 5D**), while phacoemulsification + filtering surgery resulted in a significant reduction in IOP in the short term (MD = -12.74 mmHg; 95% CI: -15.31, -10.17; Q = 0.21, p = 0.65, I^2^ = 0%) (**Figure 5D**). In the medium term, a single study showed that standalone phacoemulsification resulted in a non-significant increase in IOP (MD = 0.40 mmHg, 95% CI: -1.23, 2.03) (**Figure 5E**). Because this estimate is derived from one study, it should be interpreted cautiously and considered preliminary.

**Figure 6:**
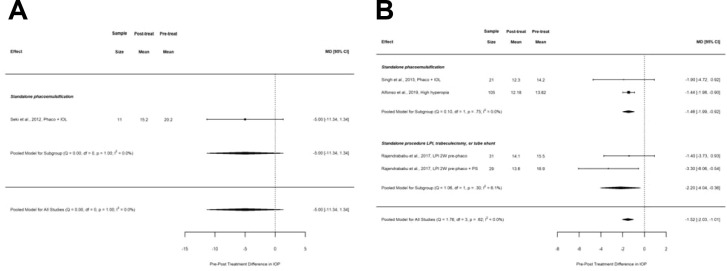
Forest plot showing the mean difference in intraocular pressure pre- and post-treatment for patients with **(A)** high hyperopia at short-term follow-up and **(B)** high hyperopia at medium-term follow-up, subgrouped by surgical technique.

Lastly, for patients with high hyperopia, a single study showed that standalone phacoemulsification resulted in a non-significant reduction in IOP in the short term (MD = -5.00 mmHg; 95% CI: -11.34, 1.34) (**Figure 6A**). Because this estimate is derived from one study, it should be interpreted cautiously and considered preliminary. In contrast, standalone phacoemulsification (MD = -1.46 mmHg; 95% CI: -1.99, -0.92; Q = 0.10, p = 0.75, I^2^ = 0%) (**Figure 6B**) and standalone LPI (MD = -2.20 mmHg, 95% CI: -4.04, -0.36; Cochran’s Q = 1.06, p = 0.30, I^2^ = 6.1%) (**Figure 6B**) both resulted in significant IOP reduction in the medium term for patients with high hyperopia.

Leave-one-out sensitivity analyses demonstrated that no individual study substantially altered the magnitude, direction, or statistical significance of the pooled estimates (**Supplementary Figure 4**).

#### Anterior chamber depth outcomes

**Figure 7:**
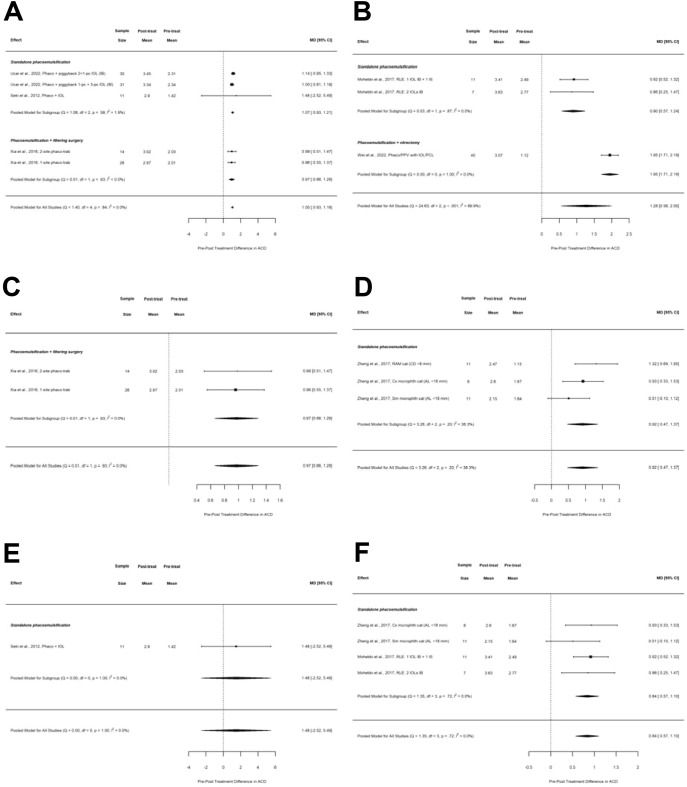
Forest plot showing the mean difference in anterior chamber depth pre- and post-treatment for patients with **(A)** nanophthalmos at short-term follow-up, **(B)** nanophthalmos at medium-term follow-up, **(C)** microphthalmos at short-term follow-up, **(D)** microphthalmos at medium-term follow-up, **(E)** high hyperopia at short-term follow-up, and **(F)** high hyperopia at medium-term follow-up, subgrouped by surgical technique.

Both standalone phacoemulsification (MD = 1.07 mm; 95% CI: 0.93, 1.21; Q = 1.08, p = 0.58, I^2^ = 1.9%) (**Figure 7A**) and phacoemulsification + filtering surgery (MD = 0.97 mm; 95% CI: 0.66, 1.29; Q = 0.01, p = 0.93, I^2^ = 0%) (**Figure 7A**) resulted in a significant increase in ACD for patients with nanophthalmos in the short term. Similarly, in the medium term, both standalone phacoemulsification (MD = 0.90 mm; 95% CI: 0.57, 1.24; Q = 0.03, p = 0.87, I^2^ = 0%) (**Figure 7B**) and a single study involving phacoemulsification + vitrectomy (MD = 1.95 mm; 95% CI: 1.71, 2.19) (**Figure 7B**) resulted in a significant increase in ACD for patients with nanophthalmos. Because this estimate is derived from one study, it should be interpreted cautiously and considered preliminary.

For patients with microphthalmos, phacoemulsification + filtering surgery resulted in a significant increase in ACD in the short term (MD = 0.97 mm; 95% CI: 0.66, 1.29; Q = 0.01, p = 0.93, I^2^ = 0%) (**Figure 7C**). Similarly, standalone phacoemulsification resulted in a significant increase in ACD in the medium term for patients with microphthalmos (MD = 0.92 mm; 95% CI: 0.47, 1.37; Q = 3.26, p = 0.20, I^2^ = 38.3%) (**Figure 7D**).

Lastly, for patients with high hyperopia, a single study showed that standalone phacoemulsification resulted in a non-significant increase in ACD in the short term (MD = 1.48 mm; 95% CI: -2.52, 5.49) (**Figure 7E**). Because this estimate is derived from one study, it should be interpreted cautiously and considered preliminary. In contrast, standalone phacoemulsification resulted in a significant increase in ACD for patients with high hyperopia in the medium term (MD = 0.84 mm; 95% CI: 0.57, 1.10; Q = 1.35, p = 0.72, I^2^ = 0%) (**Figure 7F**).

Leave-one-out sensitivity analyses demonstrated that no individual study substantially altered the magnitude, direction, or statistical significance of the pooled estimates (**Supplementary Figure 5**).

### Subgroup analyses

Subgroup analysis was performed to evaluate the effect of surgical intervention on RE, VA, IOP, and ACD stratified by disease severity according to axial length (AL < 18 mm, AL 18–20 mm, and AL 20–22 mm; with or without concomitant glaucoma).

#### Refractive error

**Figure 8:**
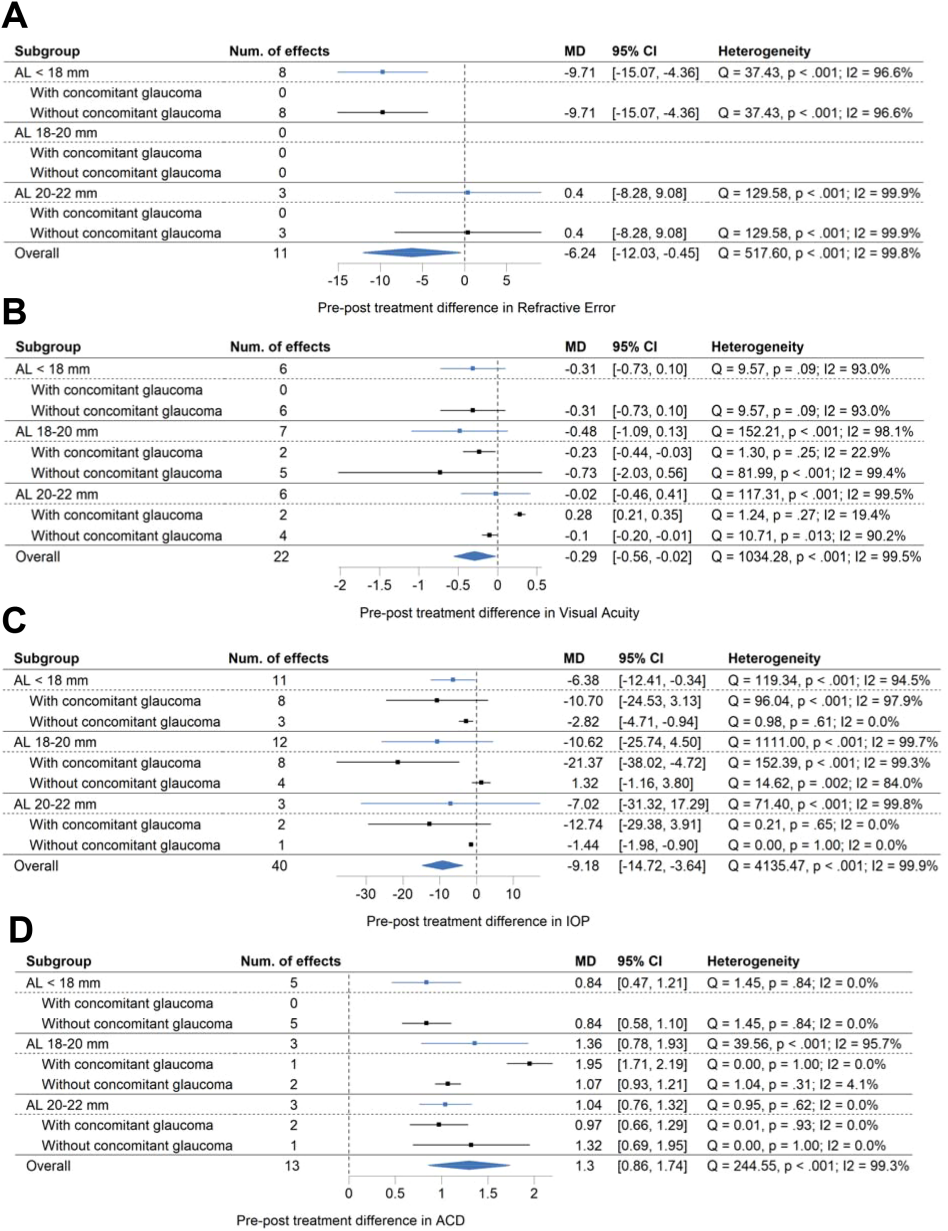
Subgroup analyses showing the effect of disease severity by axial length on **(A)** refractive error, **(B)** visual acuity, **(C)** intraocular pressure, and **(D)** anterior chamber depth.

Subgroup analysis demonstrated a significant overall reduction in RE (MD: -6.24 D; 95% CI: -12.03, -0.45; Q = 517.60, p < 0.001, I^2^ = 99.8%) (**Figure 8A**) with high heterogeneity in the effects. More specifically, this significant overall reduction in RE was largely driven by the AL < 18 mm group, which showed a significant decrease in RE (MD: -9.71 D; 95% CI: -15.07, -4.36; Q = 37.43, p < 0.001, I^2^ = 96.6%) (**Figure 8A**) with high heterogeneity in the effects. In contrast, the AL 20–22 mm group showed a non-significant change in RE (MD: 0.40 D; 95% CI: -8.28, 9.08; Q = 129.58, p < 0.001, I^2^ = 99.9%) (**Figure 8A**) with high heterogeneity in the effects. Visual inspection of the funnel plot demonstrated mild asymmetry; however, Egger’s regression test was not statistically significant (p = 0.16), suggesting no clear evidence of publication bias for RE outcomes (**Supplementary Figure 6A**).

#### Visual acuity

Subgroup analysis showed a modest but significant improvement in VA overall (MD: -0.29 logMAR; 95% CI: -0.56, -0.02; Q = 1034.28, p < 0.001, I^2^ = 99.5%) (**Figure 8B**) with high heterogeneity in the effects. Regardless of glaucoma status, the AL < 18 mm group (MD: -0.31 logMAR; 95% CI: -0.73, 0.10; Q = 9.57, p = 0.09, I^2^ = 93%) (**Figure 8B**), the AL 18–20 mm group (MD: -0.48 logMAR; 95% CI: -1.09, 0.13; Q = 152.21, p < 0.001, I^2^ = 98.1%) (**Figure 8B**), and the AL 20–22 mm group (MD: -0.02 logMAR; 95% CI: -0.46, 0.41; Q = 117.31, p < 0.001, I^2^ = 99.5%) (**Figure 8B**) overall all saw non-significant improvements in VA with high heterogeneity in the effects. Interestingly, in the AL 18–20 mm group, patients with concomitant glaucoma showed significant improvement in VA (MD: -0.23 logMAR; 95% CI: -0.44, -0.03; Q = 1.30, p = 0.25, I^2^ = 22.9%) (**Figure 8B**), while those without concomitant glaucoma showed non-significant improvement in VA (MD: -0.73 logMAR; 95% CI: -2.03, 0.56; Q = 81.99, p < 0.001, I^2^ = 99.4%) (**Figure 8B**) with high heterogeneity in the effects. Visual inspection of the funnel plot demonstrated asymmetry, and Egger’s regression test was statistically significant (p = 0.03), suggesting possible publication bias or other small-study effects for VA outcomes (**Supplementary Figure 6B**). This indicates that studies reporting larger treatment benefits may be overrepresented in the literature. Consequently, the pooled estimates for these outcomes should be interpreted with caution, as the true effect sizes may be smaller than those observed in the present meta-analysis.

#### Intraocular pressure

Subgroup analysis showed a significant overall reduction in IOP (MD: -9.18 mmHg; 95% CI: -14.72, -3.64; Q = 4135.47, p < 0.001, I^2^ = 99.9%) (**Figure 8C**) with high heterogeneity in the effects. Across all axial length subgroups, the presence of concomitant glaucoma was associated with a greater reduction in IOP compared to without concomitant glaucoma. For instance, in the AL 18–20 mm group, those with concomitant glaucoma saw a significant mean reduction in IOP (MD: -21.37 mmHg; 95% CI: -38.02, -4.72; Q = 152.39, p > 0.001, I^2^ = 99.3%) (**Figure 8C**) with high heterogeneity in the effects, whereas those without concomitant glaucoma saw a non-significant mean increase in IOP (MD: 1.32 mmHg; 95% CI: -1.16, 3.80; Q = 14.62, p = 0.002, I^2^ = 84%) (**Figure 8C**). Additionally, regardless of glaucoma status, the AL < 18 mm group overall saw a significant reduction in IOP (MD: -6.38 mmHg; 95% CI: -12.41, -0.34; Q = 119.34, p < 0.001, I^2^ = 94.5%) (**Figure 8C**) with high heterogeneity in the effects, while the AL 18–20 mm (MD: -10.62 mmHg; 95% CI: -25.74, 4.50; Q = 1111, p < 0.001, I^2^ = 99.7%) (**Figure 8C**) and AL 20–22 mm (MD: -7.02 mmHg; 95% CI: -31.32, 17.29; Q = 71.40, p < 0.001, I^2^ = 99.8%) (**Figure 8C**) groups overall saw non-significant reductions in IOP with high heterogeneity in the effects. Visual inspection of the funnel plot did not demonstrate substantial asymmetry, and Egger’s regression test was not statistically significant (p = 0.73), suggesting no evidence of publication bias for IOP outcomes (**Supplementary Figure 6C**).

#### Anterior chamber depth

Subgroup analysis showed a significant overall increase in ACD (MD: 1.30 mm; 95% CI: 0.86, 1.74; Q = 244.55, p < 0.001, I^2^ = 99.3%) (**Figure 8D**) with high heterogeneity in the effects. Across all axial length subgroups, significant ACD deepening was achieved regardless of glaucoma status. Interestingly, in the AL 18–20 mm group, patients with concomitant glaucoma from a single study showed a greater increase in ACD (MD: 1.95 mm; 95% CI: 1.71, 2.19) (**Figure 8D**) than those without concomitant glaucoma (MD: 1.07 mm; 95% CI: 0.93, 1.20; Q = 1.04, p = 0.31, I^2^ = 4.1%) (**Figure 8D**). Because this estimate is derived from one study, it should be interpreted cautiously and considered preliminary. Visual inspection of the funnel plot demonstrated asymmetry, and Egger’s regression test was statistically significant (p = 0.01), indicating possible publication bias or small-study effects for ACD outcomes (**Supplementary Figure 6D**). This indicates that studies reporting larger treatment benefits may be overrepresented in the literature. Consequently, the pooled estimates for these outcomes should be interpreted with caution, as the true effect sizes may be smaller than those observed in the present meta-analysis.

### Meta-regression

Meta-regression was performed to identify the effect of age and race on post-operative RE, VA, IOP, and ACD.

**Figure 9:**
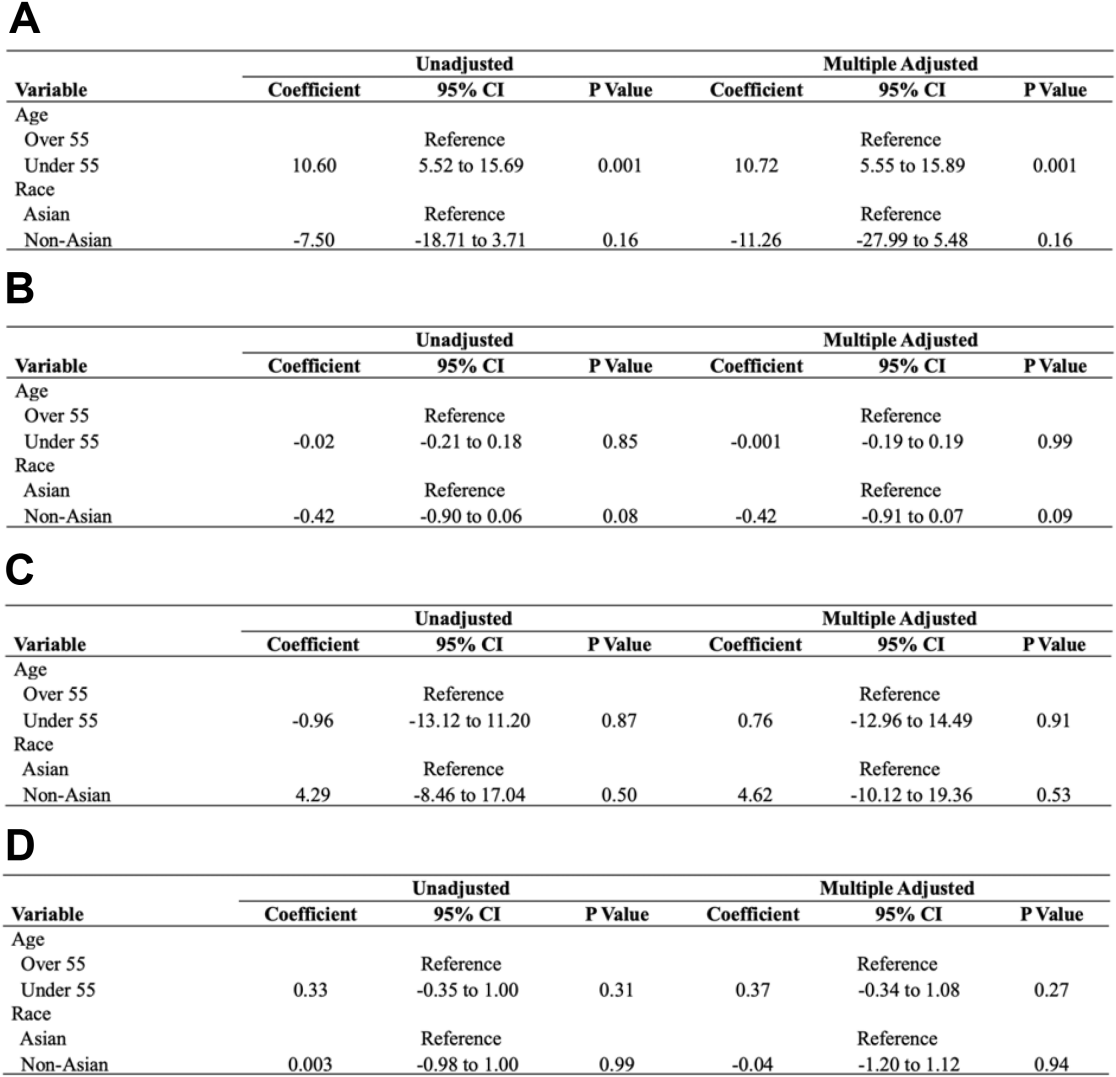
Meta-regression analysis for postoperative **(A)** refractive error, **(B)** visual acuity, **(C)** intraocular pressure, and **(D)** anterior chamber depth.

Meta-regression demonstrated a significant association between mean study age and post-operative RE (multiple adjusted coefficient: 10.72, p = 0.001) (**Figure 9**). Because this analysis was based on study-level characteristics, the finding should be interpreted as an exploratory association rather than a patient-level predictive effect. No effect of age on VA, IOP, or ACD was detectable in this study-level meta-regression (**Figure 9**). Similarly, no effect of race was detectable in this study-level meta-regression, although power was limited (**Figure 9**).

### Post-operative complications

The most frequently reported post-operative complications (**Supplementary Table 2**) include corneal edema, shallow anterior chambers, and fibrin membranes or exudation, indicating inflammation. Posterior capsule opacification (PCO) and transient IOP elevation also appeared repeatedly across different types of cataract and glaucoma surgeries. The most serious and sight-threatening complications were retinal detachment, suprachoroidal hemorrhage, and malignant glaucoma (aqueous misdirection). Severe uveal effusion and iatrogenic retinal holes were also highlighted as critical complications that compromise the structural integrity and long-term health of the eye.

## Discussion

This systematic review and meta-analysis represents a comprehensive synthesis of surgical outcomes in adult (≥ 18 years) patients with nanophthalmos, microphthalmos, and high hyperopia. The findings suggest that surgical intervention is associated with improvements in RE, VA, IOP, and ACD in these challenging eyes, with outcomes varying substantially based on surgical technique and patient characteristics.

### Refractive error

Standalone phacoemulsification appeared to produce a meaningful reduction in RE for patients with nanophthalmos at both short-term (MD = -5.70 D) and medium-term (MD = -13.20 D) follow-up, as well as for patients with high hyperopia at both short-term (MD = -3.68 D) and medium-term (MD = -9.23 D) follow-up, though these estimates should be interpreted cautiously given the predominantly non-randomized evidence base and moderate overall risk of bias. These trends are broadly consistent with prior observational data; for example, Singh et al. reported that spherical equivalent improved from +16.11 D preoperatively to +2.00 D postoperatively (p < 0.0001) in nanophthalmic eyes implanted with high-power foldable IOLs, though this too was a non-randomized study subject to similar limitations (19). In contrast, standalone phacoemulsification was associated with a non-significant change in refractive error for patients with microphthalmos at medium-term follow-up (MD = -0.34 D; I² = 95.5%), with high heterogeneity likely driven by divergent effects across microphthalmic subtypes – patients with relative anterior microphthalmos and small corneal diameters experienced a hyperopic shift, while those with simple and complex microphthalmos experienced a shift in the opposite direction. This subtype-dependent variability is consistent with observations by Jung et al., who noted that refractive predictability tended to be poorer in nanophthalmic eyes compared to eyes with relative anterior microphthalmos, with IOL power formulas yielding greater mean absolute errors in the shortest eyes (41). Similarly, Singh et al. reported that the Hoffer Q formula achieved a final refraction within 1.0 D of target in only 61% of small eyes, further highlighting the persistent challenge of IOL power calculation in this population (42). Subgroup analysis suggested that the overall reduction in refractive error (MD = -6.24 D) was largely driven by the AL < 18 mm group (MD = -9.71 D), while the AL 20–22 mm group showed a non-significant change (MD = 0.40 D). Meta-regression identified a significant association between mean study age and post-operative RE (coefficient = 10.72, p = 0.001); however, because meta-regression utilizes aggregate study-level data rather than individual patient data, this finding should be interpreted cautiously. The observed association may reflect differences between studies rather than a true patient-level effect and is therefore susceptible to ecological fallacy. Additionally, age may be correlated with other study characteristics that could not be accounted for in the present analysis, including baseline RE, ocular biometry, IOL power calculation methods, and surgeon experience.

### Visual acuity

Standalone phacoemulsification was associated with an improvement in visual acuity for patients with nanophthalmos at both short-term (MD = -0.66 logMAR) and medium-term (MD = -0.70 logMAR) follow-up, though the high heterogeneity and predominantly observational study designs limit the certainty of these pooled estimates. These trends are broadly consistent with the observational findings of Yosar et al., who reported that phacoemulsification improved corrected distance visual acuity in 74.6% of short and nanophthalmic eyes, with 66.2% achieving logMAR 0.30 or better (43). Phacoemulsification + vitrectomy was also associated with a medium-term improvement in visual acuity for nanophthalmic patients (MD = -0.23 logMAR; I² = 22.9%), a finding that aligns with the observational data of Wei et al., who reported that combined limited pars plana vitrectomy and phacoemulsification was associated with improved logMAR BCVA from 1.28 to 0.96 (p < 0.001) in complex nanophthalmos (44). Notably, phacoemulsification + filtering surgery was associated with a short-term worsening of visual acuity in both nanophthalmic (MD = +0.28 logMAR) and microphthalmic (MD = +0.28 logMAR) cohorts, a finding that may reflect the additional surgical trauma and postoperative inflammation associated with combined glaucoma procedures in these anatomically challenging eyes, though confounding by indication – whereby patients with more severe disease are more likely to undergo combined procedures – cannot be excluded in the absence of randomized data. Fan et al. similarly observed that filtering surgery alone in nanophthalmic eyes with angle-closure glaucoma was associated with a high complication rate and less visual improvement compared to combined approaches, though this comparison was also non-randomized (45). For patients with high hyperopia, standalone phacoemulsification was associated with a significant short-term improvement in visual acuity (MD = -0.06 logMAR) but a non-significant medium-term improvement (MD = -0.31 logMAR; I² = 92.8%), though all seven medium-term studies reported effects in the same direction, suggesting a possible consistent trend toward visual improvement despite the high statistical heterogeneity.

### Intraocular pressure

Intraocular pressure outcomes varied substantially by surgical approach and disease subtype, though the predominantly non-randomized designs and high heterogeneity across many pooled estimates warrant cautious interpretation. For nanophthalmic patients, phacoemulsification + vitrectomy was associated with the largest IOP reductions, with point estimates suggesting decreases at short-term (MD = -19.20 mmHg), medium-term (MD = -21.03 mmHg), and long-term (MD = -17.06 mmHg) follow-up, while standalone phacoemulsification was associated with non-significant IOP reductions at both short-term (MD = -5.00 mmHg) and medium-term (MD = -1.90 mmHg). These observations are broadly concordant with Wei et al., who reported a mean IOP decrease from 32.7 to 16.9 mmHg (p < 0.001) after combined vitrectomy-phacoemulsification in complex nanophthalmos, and with Fan et al., who observed that triple-procedure surgery achieved lower late-stage IOP (13.29 mmHg) compared to filtering surgery alone (27.57 mmHg), though neither study was randomized (44, 45). The apparent difference in IOP-lowering effect between combined and standalone procedures may partly reflect confounding by indication, as patients undergoing combined procedures likely had higher baseline IOP and more advanced glaucoma. The modest IOP-lowering effect of standalone phacoemulsification observed in the present meta-analysis is broadly consistent with large registry data, which have suggested a mean IOP decrease of approximately 1.5 mmHg after standalone phacoemulsification across all eyes, though the IOP-lowering effect of lens extraction may be more pronounced in angle-closure disease (19, 28). Subgroup analysis suggested that concomitant glaucoma was associated with greater IOP reduction across axial length groups; in the AL 18–20 mm subgroup, patients with glaucoma experienced a mean IOP reduction of -21.37 mmHg compared to a non-significant increase of +1.32 mmHg in those without glaucoma, though this difference may again reflect higher baseline IOP in the glaucoma group rather than a differential treatment effect. For patients with high hyperopia, standalone phacoemulsification and standalone LPI were both associated with modest medium-term IOP reductions (MD = -1.46 mmHg and -2.20 mmHg, respectively), consistent with the proposed role of lens extraction and LPI in managing angle-closure mechanisms in hyperopic eyes (28, 45).

### Anterior chamber depth

Both standalone phacoemulsification and combined surgical approaches appeared to produce anterior chamber deepening across all disease subtypes, though the magnitude of these estimates should be interpreted in the context of the predominantly observational evidence. For nanophthalmic patients, standalone phacoemulsification was associated with an ACD increase of 1.07 mm in the short-term and 0.90 mm in the medium-term, while phacoemulsification + vitrectomy was associated with the greatest ACD increase (1.95 mm at medium-term follow-up from a single study). These observations are consistent with Seki et al., who reported that phacoemulsification deepened the anterior chamber and widened the anterior chamber angle in nanophthalmic eyes with a mean axial length of 17.3 mm, and with Wei et al., who observed a mean ACD increase from 1.14 mm to 3.07 mm (p < 0.001) after phacoemulsification + vitrectomy (44, 46). The magnitude of ACD deepening observed in the present meta-analysis appears to be greater than that typically reported in eyes with normal axial lengths; Muzyka-Woźniak and Ogar found that the relative change in ACD after phacoemulsification was 57% in short eyes compared to 44% in normal eyes and 42% in long eyes, likely reflecting the disproportionately large lens-to-eye volume ratio in short eyes (47). For patients with microphthalmos, ACD deepening was observed with both phacoemulsification + filtering surgery in the short-term (MD = 0.97 mm) and standalone phacoemulsification in the medium-term (MD = 0.92 mm). For patients with high hyperopia, standalone phacoemulsification was associated with a significant medium-term ACD increase of 0.84 mm but a non-significant short-term increase (MD = 1.48 mm; 95% CI: -2.52, 5.49), likely reflecting the wide confidence interval from a single study. Subgroup analysis suggested consistent ACD deepening across all axial length categories regardless of glaucoma status, with the greatest increase observed in the AL 18–20 mm group with concomitant glaucoma (MD = 1.95 mm). While this consistent anterior chamber deepening may have implications for reducing the risk of angle-closure mechanisms and contributing to IOP-lowering effects after lens extraction in these anatomically predisposed eyes, prospective controlled studies are needed to confirm these associations and clarify the causal mechanisms involved (41, 46).

### Limitations

This systematic review and meta-analysis has several important limitations. Publication bias was detected for subgroup analyses of VA and ACD outcomes, as demonstrated by significant Egger’s regression tests and asymmetric funnel plots. These findings suggest that studies reporting larger improvements may be more likely to have been published, which could result in overestimation of the pooled treatment effects. Therefore, although the observed improvements in VA and ACD were statistically significant, the magnitude of these benefits should be interpreted cautiously. From a clinical perspective, the direction of effect is likely preserved, but the true degree of improvement may be smaller than estimated. Additional prospective studies and publication of negative or neutral findings will be important to better define the effectiveness of these interventions.

Second, the included studies were predominantly non-randomized studies with small sample sizes, inherent selection bias, and lack of standardized protocols. Third, substantial heterogeneity existed in patient baseline characteristics and surgical techniques, limiting the ability to draw specific conclusions about individual surgical approaches. Fourth, follow-up duration varied considerably across studies, ranging from 3 months to over 2 years, potentially introducing time-dependent bias in outcome assessment. Long-term outcomes, particularly regarding glaucoma progression and the development of posterior capsular opacification, were not adequately captured. Fifth, the meta-analysis relied on study-level rather than individual patient data, precluding detailed subgroup analyses and adjustment for potential confounders such as pre-operative glaucoma severity, lens thickness, and concurrent medications (48). Sixth, the lack of standardized outcome reporting across studies, particularly regarding complications and quality of life measures, prevented comprehensive synthesis of these important endpoints (49). Seventh, with 21 study-level data points, the power to detect demographic effects was very limited. Eighth, most studies originated from specialized tertiary centers with experienced surgeons, possibly limiting generalizability to community practice settings. Lastly, inclusion criteria restricted to English-language studies is a publication and language bias.

### Practice considerations beyond synthesized evidence

Based on the synthesized evidence, the following clinical guidance is proposed for surgical management of nanophthalmos, microphthalmos, and high hyperopia.

Lens-based surgery should be considered in patients with nanophthalmos, microphthalmos, or high hyperopia who have visually significant refractive error, angle closure disease, or cataract. Early intervention with phacoemulsification is preferable to delayed surgery requiring manual extraction techniques, as complication rates are significantly lower (20). Patients should be counseled that while visual and anatomical outcomes are generally favorable, short eyes carry higher surgical risks than routine cataract surgery, with complication rates of 15-28% (19, 21, 28).

Comprehensive pre-operative evaluation should include optical biometry with measurement of axial length, anterior chamber depth, lens thickness, and corneal diameter. Ultrasound biomicroscopy or anterior segment optical coherence tomography (OCT) can assess angle configuration and identify peripheral anterior synechiae (34, 50). Brightness-scan ultrasonography should measure retinal-choroidal-scleral thickness, as values >2.0 mm confirm nanophthalmos and predict higher complication risk (25). Gonioscopy is essential to document angle closure extent and guide surgical planning.

The Haigis, Kane, or Barrett Universal II formulas should be used for IOL power calculation in nanophthalmic, microphthalmic, and highly hyperopic eyes, with lens constants optimized for short axial lengths (51–55). Surgeons should inform patients about potential refractive surprises and the possible need for secondary procedures. In extreme cases requiring IOL powers beyond available ranges, piggyback IOL implantation (one in the capsular bag, one in the sulcus) represents a viable option (56).

For nanophthalmic eyes with angle closure glaucoma and IOP elevation, combined procedures incorporating phacoemulsification with goniosynechialysis, limited vitrectomy, and/or posterior capsulotomy provide superior IOP control compared to phacoemulsification alone (47, 57, 58). The specific combination should be tailored to individual anatomy, with goniosynechialysis reserved for eyes with documented peripheral anterior synechiae, and vitrectomy considered for eyes with shallow anterior chambers or risk of aqueous misdirection (11, 59). Prophylactic sclerostomy should be considered in eyes with axial length 17 mm, thick sclera (>2.0 mm), or history of uveal effusion, though routine use in all nanophthalmic eyes remains controversial (14, 20, 60). Intraoperative measures to prevent complications include pre-operative systemic mannitol or acetazolamide to reduce vitreous volume, generous use of cohesive viscoelastics to maintain anterior chamber depth, and meticulous wound construction to prevent hypotony (11).

Close monitoring for complications including uveal effusion, aqueous misdirection, and IOP spikes is essential in the early post-operative period. Topical corticosteroids should be used liberally to prevent inflammation-related complications. Patients should be counseled about the potential need for additional procedures, including yttrium aluminum garnet (YAG) capsulotomy, refractive enhancement, or glaucoma surgery.

## Conclusion

This systematic review and meta-analysis demonstrates that surgical interventions for adults (≥ 18 years) with nanophthalmos, microphthalmos, and high hyperopia can provide meaningful improvements in refractive, visual, anatomical, and IOP outcomes, although treatment response varies according to underlying ocular anatomy, axial length, glaucoma status, and surgical approach. Standalone phacoemulsification consistently resulted in improved RE, VA, and ACD, across multiple patient groups, while combined procedures, particularly phacoemulsification + vitrectomy or phacoemulsification + filtering surgery, achieved greater IOP reduction in eyes with concomitant glaucoma or more advanced angle-closure disease. Subgroup analyses further suggest that eyes with shorter axial lengths may experience greater refractive and pressure-lowering benefits, whereas the presence of concomitant glaucoma appears to strongly influence post-operative IOP outcomes. Despite these favorable findings, substantial heterogeneity across studies highlights the complexity of managing these anatomically challenging eyes and underscores the influence of variations in surgical technique, baseline disease severity, and follow-up duration. Importantly, post-operative complications – including shallow anterior chamber, uveal effusion, malignant glaucoma, retinal detachment, and suprachoroidal hemorrhage – remain significant concerns and emphasize the need for meticulous perioperative planning and individualized surgical decision-making. Given the predominance of retrospective, non-randomized studies and limited long-term data, future prospective multicenter studies with standardized outcome reporting are needed to better define optimal surgical strategies and improve risk stratification in this high-risk population.

## Data Availability

The original contributions presented in the study are included in the article/**Supplementary Material**. Further inquiries can be directed to the corresponding author.

## Glossary

1-site phaco-trab: 1-site phacoemulsification combined with trabeculectomy
2-site phaco-trab: 2-site phacoemulsification combined with trabeculectomy
23G PPV/PPL: 23-gauge pars plana vitrectomy combined with lensectomy
AL: Axial length
CD: Corneal diameter
Cx microphth cat: Cataract of complex microphthalmos
Low-mod hyperopia: Low-moderate hyperopia
LPI 2W pre-phaco: Laser peripheral iridotomy 2 weeks prior to cataract surgery
LPI 2W pre-phaco + PS: Laser peripheral iridotomy 2 weeks prior to cataract surgery with concomitant prophylactic sclerostomy
Phaco + ant vit: Phacoemulsification with anterior vitrectomy
Phaco + ant vit + sclerotomy: Phacoemulsification with anterior vitrectomy and sclerotomy
Phaco + IOL: Phacoemulsification with intraocular lens implantation
Phaco + piggyback 1-pc + 3-pc IOL (IB): Phacoemulsification with piggyback implantation of a one-piece intraocular lens along with a three-piece intraocular lens into the capsular bag
Phaco + piggyback 2x1-pc IOL (IB): Phacoemulsification with piggyback implantation of two one-piece intraocular lenses into the capsular bag
Phaco/PPV with IOL/PCL: Limited pars plana vitrectomy + phacoemulsification with intraocular lens implantation + posterior capsulotomy
RAM cat: Cataract of relative anterior microphthalmos
RLE: 1 IOL IB + 1 IS: Refractive lens exchange with implantation of 1 intraocular lens in bag and 1 in sulcus
RLE: 2 IOLs IB: Refractive lens exchange with implantation of both intraocular lenses in bag
Sm microphth cat: Cataract of simple microphthalmos
Surgical tx for UG: Surgical treatment for uncontrolled glaucoma (trabeculectomy, trabeculectomy + sclerostomy, trabeculectomy + intraocular lens + sclerostomy).

## References

[1] Awadalla MS Burdon KP Souzeau E Landers J Hewitt AW Sharma S . Mutation in TMEM98 in a large white kindred with autosomal dominant nanophthalmos linked to 17p12-q12. JAMA Ophthalmol. (2014) 132:970–7. doi: 10.1001/jamaophthalmol.2014.946 24852644

[2] Hassall MM Javadiyan S Klebe S Awadalla MS Sharma S Qassim A . Phenotypic consequences of a nanophthalmos-associated TMEM98 variant in human and mouse. Sci Rep. (2023) 13:11017. doi: 10.1038/s41598-023-37855-x 37419942 PMC10328987

[3] Yu X Zhao H Gao Y Zhou T Deng L Zhang M . Genetic spectrum and genotype-phenotype correlations in a Chinese cohort with nanophthalmos with secondary angle-closure glaucoma. Invest Ophthalmol Vis Sci. (2025) 66:9. doi: 10.1167/iovs.66.6.9 40459495 PMC12136104

[4] Fernández-Vigo JI Rodríguez-Quet O Montolío-Marzo E Burgos-Blasco B Kudsieh B González-Martin-Moro J . Anterior scleral thickness and other dimensions in nanophthalmos by swept-source optical coherence tomography: a comparative study. J Clin Med. (2023) 12:5564. doi: 10.3390/jcm12175564 37685634 PMC10488421

[5] Li X Xiao H Su Y Xiao X Li S Lin S . Clinical features of patients with mutations in genes for nanophthalmos. Br J Ophthalmol. (2024) 108:1679–87. doi: 10.1136/bjo-2023-324931 38749530

[6] Othman MI Sullivan SA Skuta GL Cockrell DA Stringham HM Downs CA . Autosomal dominant nanophthalmos (NNO1) with high hyperopia and angle-closure glaucoma maps to chromosome 11. Am J Hum Genet. (1998) 63:1411–8. doi: 10.1086/302113 9792868 PMC1377551

[7] Jackson D Moosajee M . The genetic determinants of axial length: from microphthalmia to high myopia in childhood. Annu Rev Genomics Hum Genet. (2023) 24:177–202. doi: 10.1146/annurev-genom-102722-090617 37624667

[8] Skalicky SE White AJR Grigg JR Martin F Smith J Jones M . Microphthalmia, anophthalmia, and coloboma and associated ocular and systemic features: understanding the spectrum. JAMA Ophthalmol. (2013) 131:1517–24. doi: 10.1001/jamaophthalmol.2013.5305 24177921

[9] Alkatan HM Bedaiwi KM Al-Faky YH Maktabi AMY . Demographics and histopathological characteristics of enucleated microphthalmic globes. Sci Rep. (2022) 12:5283. doi: 10.1038/s41598-022-09261-2 35347187 PMC8960817

[10] Prasov L Guan B Ullah E Archer SM Ayres BM Besirli CG . Novel TMEM98, MFRP, PRSS56 variants in a large United States high hyperopia and nanophthalmos cohort. Sci Rep. (2020) 10:19986. doi: 10.1038/s41598-020-76725-8 33203948 PMC7672112

[11] Gedde SJ Chen PP Muir KW Vinod K Lind JT Wright MM . Primary angle-closure disease preferred practice pattern®. Ophthalmology. (2021) 128:P30–70. doi: 10.1016/j.ophtha.2020.10.021 34933744

[12] Relhan N Jalali S Pehre N Rao HL Manusani U Bodduluri L . High-hyperopia database, part I: clinical characterisation including morphometric (biometric) differentiation of posterior microphthalmos from nanophthalmos. Eye (Lond). (2016) 30:120–6. doi: 10.1038/eye.2015.206 26493039 PMC4709547

[13] Demircan A Altan C Osmanbasoglu OA Celik U Kara N Demirok A . Subfoveal choroidal thickness measurements with enhanced depth imaging optical coherence tomography in patients with nanophthalmos. Br J Ophthalmol. (2014) 98:345–9. doi: 10.1136/bjophthalmol-2013-303465 24307716

[14] Rajendrababu S Shroff S Uduman MS Babu N . Clinical spectrum and treatment outcomes of patients with nanophthalmos. Eye (Lond). (2021) 35:825–30. doi: 10.1038/s41433-020-0971-4 32461562 PMC8027642

[15] Rajendrababu S Shroff S More S Radhakrishnan S Chidambaranathan G Senthilkumar VA . A report on a series of nanophthalmos with histopathology and immunohistochemistry analyses using light microscopy. Indian J Ophthalmol. (2022) 70:2597–602. doi: 10.4103/ijo.IJO_2973_21 35791166 PMC9426058

[16] Elagouz M Stanescu-Segall D Jackson TL . Uveal effusion syndrome. Surv Ophthalmol. (2010) 55:134–45. doi: 10.1016/j.survophthal.2009.05.003 20159229

[17] Zhou N Yang L Xu X Wei W . Uveal effusion syndrome: clinical characteristics, outcome of surgical treatment, and histopathological examination of the sclera. Front Med (Lausanne). (2022) 9:785444. doi: 10.3389/fmed.2022.785444 35755052 PMC9218343

[18] Amer R Nalcı H Yalçındağ N . Exudative retinal detachment. Surv Ophthalmol. (2017) 62:723–69. doi: 10.1016/j.survophthal.2017.05.001 28506603

[19] Yosar JC Zagora SL Grigg JR . Cataract surgery in short eyes, including nanophthalmos: visual outcomes, complications and refractive results. Clin Ophthalmol. (2021) 15:4543–51. doi: 10.2147/OPTH.S344465 34866899 PMC8636843

[20] Rajendrababu S Babu N Sinha S Balakrishnan V Vardhan A Puthuran GV . A randomized controlled trial comparing outcomes of cataract surgery in nanophthalmos with and without prophylactic sclerostomy. Am J Ophthalmol. (2017) 183:125–33. doi: 10.1016/j.ajo.2017.09.008 28911991

[21] Steijns D Bijlsma WR Van der Lelij A . Cataract surgery in patients with nanophthalmos. Ophthalmology. (2013) 120:266–70. doi: 10.1016/j.ophtha.2012.07.082 23084128

[22] Hoffman RS Vasavada AR Allen QB Snyder ME Devgan U Braga-Mele R . Cataract surgery in the small eye. J Cataract Refract Surg. (2015) 41:2565–75. doi: 10.1016/j.jcrs.2015.10.008 26703508

[23] Chronopoulos A Thumann G Schutz J . Positive vitreous pressure: pathophysiology, complications, prevention, and management. Surv Ophthalmol. (2017) 62:127–33. doi: 10.1016/j.survophthal.2016.10.002 27751822

[24] Alkharashi M AlAbdulhadi HA Otaif W Alahmadi AS Alanazi B Al Habash A . Incidence, pathophysiology, complications, and management of positive vitreous pressure during penetrating keratoplasty: a literature review. Clin Ophthalmol. (2023) 17:583–90. doi: 10.2147/OPTH.S382502 36820300 PMC9938663

[25] Wu W Dawson DG Sugar A Elner SG Meyer KA McKey JB . Cataract surgery in patients with nanophthalmos: results and complications. J Cataract Refract Surg. (2004) 30:584–90. doi: 10.1016/j.jcrs.2003.07.009 15050253

[26] Chan FM Lee L . Nanophthalmic cataract extraction. Clin Exp Ophthalmol. (2004) 32:535–8. doi: 10.1111/j.1442-9071.2004.00873.x 15498069

[27] Calhoun FP . The management of glaucoma in nanophthalmos. Trans Am Ophthalmol Soc. (1975) 73:97–122. 1246819 PMC1311447

[28] Day AC MacLaren RE Bunce C Stevens JD Foster PJ . Outcomes of phacoemulsification and intraocular lens implantation in microphthalmos and nanophthalmos. J Cataract Refract Surg. (2013) 39:87–96. doi: 10.1016/j.jcrs.2012.08.057 23245362

[29] Yalvac IS Satana B Ozkan G Eksioglu U Duman S . Management of glaucoma in patients with nanophthalmos. Eye (Lond). (2008) 22:838–43. doi: 10.1038/sj.eye.6702742 17293784

[30] Bao YK Xu BY Friedman DS Cho A Foster PJ Jiang Y . Biometric risk factors for angle closure progression after laser peripheral iridotomy. JAMA Ophthalmol. (2023) 141:516–24. doi: 10.1001/jamaophthalmol.2023.0937 37103926 PMC10141278

[31] Lim R . The surgical management of glaucoma: a review. Clin Exp Ophthalmol. (2022) 50:213–31. doi: 10.1111/ceo.14028 35037376

[32] Sunaric Megevand G Bron AM . Personalising surgical treatments for glaucoma patients. Prog Retin Eye Res. (2021) 81:100879. doi: 10.1016/j.preteyeres.2020.100879 32562883

[33] Costa VP Leung CKS Kook MS Lin SC Global Glaucoma Academy . Clear lens extraction in eyes with primary angle closure and primary angle-closure glaucoma. Surv Ophthalmol. (2020) 65:662–74. doi: 10.1016/j.survophthal.2020.04.003 32339525

[34] Seki M Fukuchi T Ueda J Suda K Nakatsue T Tanaka Y . Nanophthalmos: quantitative analysis of anterior chamber angle configuration before and after cataract surgery. Br J Ophthalmol. (2012) 96:1108–16. doi: 10.1136/bjophthalmol-2012-301496 22661652

[35] Ally N Ismail S Alli HD . Prevalence of complications in eyes with nanophthalmos or microphthalmos: protocol for a systematic review and meta-analysis. Syst Rev. (2022) 11:25. doi: 10.1186/s13643-022-01889-5 35139896 PMC8829984

[36] Page MJ McKenzie JE Bossuyt PM Boutron I Hoffmann TC Mulrow CD . The PRISMA 2020 statement: an updated guideline for reporting systematic reviews. BMJ. (2021) 372:n71. doi: 10.1136/bmj.n71 33782057 PMC8005924

[37] Kottner J Audigé L Brorson S Donner A Gajewski BJ Hróbjartsson A . Guidelines for reporting reliability and agreement studies (GRRAS) were proposed. J Clin Epidemiol. (2011) 64:96–106. doi: 10.1016/j.jclinepi.2010.03.002 21130355

[38] Sterne JAC Savović J Page MJ Elbers RG Blencowe NS Boutron I . RoB 2: a revised tool for assessing risk of bias in randomised trials. BMJ. (2019) 366:l4898. doi: 10.1136/bmj.l4898 31462531

[39] Sterne JA Hernán MA Reeves BC Savović J Berkman ND Viswanathan M . ROBINS-I: a tool for assessing risk of bias in non-randomised studies of interventions. BMJ. (2016) 355:i4919. doi: 10.1136/bmj.i4919 27733354 PMC5062054

[40] Harrer M Cuijpers P Furukawa T Ebert D . Doing Meta-Analysis With R: A Hands-on Guide. New York, United States: Chapman and Hall/CRC (2021). doi: 10.1201/9781003107347

[41] Lai THT Tse JYT Chan JWT Li KKK . Visual and refractive outcomes after phacoemulsification cataract surgery in nanophthalmic eyes. J Clin Med. (2024) 13:5852. doi: 10.3390/jcm13195852 39407912 PMC11477411

[42] Singh H Wang JCC Desjardins DC Baig K Gagné S Ahmed IIK . Refractive outcomes in nanophthalmic eyes after phacoemulsification and implantation of a high-refractive-power foldable intraocular lens. J Cataract Refract Surg. (2015) 41:2394–402. doi: 10.1016/j.jcrs.2015.05.033 26703488

[43] Jung KI Yang JW Lee YC Kim SY . Cataract surgery in eyes with nanophthalmos and relative anterior microphthalmos. Am J Ophthalmol. (2012) 153:1161–1168.e1. doi: 10.1016/j.ajo.2011.12.006 22365256

[44] Zheng T Chen Z Xu J Tang Y Fan Q Lu Y . Outcomes and prognostic factors of cataract surgery in adult extreme microphthalmos with axial length <18 mm or corneal diameter <8 mm. Am J Ophthalmol. (2017) 184:84–96. doi: 10.1016/j.ajo.2017.09.028 28988897

[45] Zhu H Sun C Xiao R Song Z Shu X Gong Y . Clinical management of nanophthalmos combined with cataract: a case series. Front Med (Lausanne). (2026) 13:1827475. doi: 10.3389/fmed.2026.1827475 42221105 PMC13216057

[46] Rajendrababu S Berendchot TTJM Hariyani R Prasad S Sundar B Ramya G . Comparing the surgical outcomes of femto laser-assisted cataract surgery (FLACS) versus conventional phacoemulsification (CP) in nanophthalmic eyes. Indian J Ophthalmol. (2026) 74:29–36. doi: 10.4103/IJO.IJO_18_25 41460126 PMC12867309

[47] Wei Y Su Y Fang L Guo X Chen S Han Y . Effect of combined surgery in patients with complex nanophthalmos. J Clin Med. (2022) 11:5909. doi: 10.3390/jcm11195909 36233776 PMC9571930

[48] McCullough P Mohite A Virgili G Lois N . Outcomes and complications of pars plana vitrectomy for tractional retinal detachment in people with diabetes: a systematic review and meta-analysis. JAMA Ophthalmol. (2023) 141:186–95. doi: 10.1001/jamaophthalmol.2022.5817 36633878 PMC9857853

[49] Saldanha IJ Lindsley K Do DV Chuck RS Meyerle C Jones LS . Comparison of clinical trial and systematic review outcomes for the 4 most prevalent eye diseases. JAMA Ophthalmol. (2017) 135:933–40. doi: 10.1001/jamaophthalmol.2017.2583 28772305 PMC5625342

[50] Guo C Zhao Z Zhang D Liu J Li J Zhang J . Anterior segment features in nanophthalmos with secondary chronic angle closure glaucoma: an ultrasound biomicroscopy study. Invest Ophthalmol Vis Sci. (2019) 60:2248–56. doi: 10.1167/iovs.19-26867 31112609

[51] MacLaren RE Natkunarajah M Riaz Y Bourne RRA Restori M Allan BDS . Biometry and formula accuracy with intraocular lenses used for cataract surgery in extreme hyperopia. Am J Ophthalmol. (2007) 143:920–31. doi: 10.1016/j.ajo.2007.02.043 17524766

[52] Lin P Xu J Miao A Xu C Qian D Lu Y . A comparative study on the accuracy of IOL calculation formulas in nanophthalmos and relative anterior microphthalmos. Am J Ophthalmol. (2023) 245:61–9. doi: 10.1016/j.ajo.2022.08.023 36084681

[53] Zhang G Ma Y Liu Z Jin L Zheng D Zhang X . IOL power calculation formula accuracy in 1178 eyes with short axial length: systematic reviews and network meta-analysis. J Cataract Refract Surg. (2025) 52:202–7. doi: 10.1097/j.jcrs.0000000000001793 40971912

[54] Kane JX Chang DF . Intraocular lens power formulas, biometry, and intraoperative aberrometry: a review. Ophthalmology. (2021) 128:e94–e114. doi: 10.1016/j.ophtha.2020.08.010 32798526

[55] Wendelstein J Hoffmann P Hirnschall N Fischinger IR Mariacher S Wingert T . Project hyperopic power prediction: accuracy of 13 different concepts for intraocular lens calculation in short eyes. Br J Ophthalmol. (2022) 106:795–801. doi: 10.1136/bjophthalmol-2020-318272 33504489

[56] Mohebbi M Fallah-Tafti MR Fadakar K Katoozpour R Mohammadi SF Fallah-Tafti Z . Refractive lens exchange and piggyback intraocular lens implantation in nanophthalmos: visual and structural outcomes. J Cataract Refract Surg. (2017) 43:1190–6. doi: 10.1016/j.jcrs.2017.06.038 28991616

[57] Husain R Do T Lai J Kitnarong N Nongpiur ME Perera SA . Efficacy of phacoemulsification alone vs phacoemulsification with goniosynechialysis in patients with primary angle-closure disease: a randomized clinical trial. JAMA Ophthalmol. (2019) 137:1107–13. doi: 10.1001/jamaophthalmol.2019.2493 31294768 PMC6624811

[58] Fan X Wang J Sheng Q Zhai R Kong X . Outcomes of combined phacoemulsification, anterior vitrectomy, and sclerectomy in nanophthalmic eyes with glaucoma. Eye (Lond). (2023) 37:751–9. doi: 10.1038/s41433-022-02039-w 35383309 PMC9998427

[59] Wang Y Liang ZQ Zhang Y Hennein L Han Y Wu HJ . Efficacy and safety of phacoemulsification plus goniosynechialysis and trabectome in patients with primary angle-closure glaucoma. Sci Rep. (2021) 11:13921. doi: 10.1038/s41598-021-92972-9 34230569 PMC8260581

[60] Braga de Sousa L Barbosa-Breda J . Sclerectomies in nanophthalmos and idiopathic uveal effusion syndrome: a systematic review. Graefes Arch Clin Exp Ophthalmol. (2025) 263:2709–22. doi: 10.1007/s00417-025-06908-4 40663118 PMC12583281

