# Supplementary material for: Cataract and glaucoma surgery in microphthalmic, nanophthalmic, and high hyperopic eyes: a systematic review and meta-analysis
Source: Front Ophthalmol (Lausanne). 2026 Jul 14;6:1872292. doi: 10.3389/fopht.2026.1872292 (PMC13407367; doi:10.3389/fopht.2026.1872292)
Supplement: Supplementary file 1 [file DataSheet1.docx]

**Supplemental Table 1.** Search strategy.

| **Cochrane Library** | ((nanophthalmos):ti,ab,kw OR #1 OR (microphthalmos):ti,ab,kw OR (“angle closure”):ti,ab,kw OR (“plateau iris”):ti,ab,kw OR (“phacomorphic glaucoma”):ti,ab,kw OR (“lens-induced glaucoma”):ti,ab,kw OR #2 OR (“narrow angles”):ti,ab,kw OR (“malignant glaucoma”):ti,ab,kw OR (“aqueous misdirection”):ti,ab,kw) AND #3 AND ( #4 OR (lpi):ti,ab,kw OR (“laser peripheral iridotomy”):ti,ab,kw OR #5 OR (surgery):ti,ab,kw OR (migs):ti,ab,kw OR (procedure):ti,ab,kw OR #6 OR (“laser goniotomy”):ti,ab,kw OR (gonioplasty):ti,ab,kw) AND ( #7 OR (iop):ti,ab,kw OR #8 OR #9 OR (“degree of hyperopia”):ti,ab,kw OR (“grade of angle”):ti,ab,kw OR (effectiveness):ti,ab,kw OR (“number of medications”):ti,ab,kw OR #10 OR (“anterior chamber depth”):ti,ab,kw) |
| --- | --- |
| **PubMed** | (((((((((((("Cataract Extraction"[Mesh]) AND "Adult"[Mesh]) AND ( "Nanophthalmos 1" [Supplementary Concept] OR "Nanophthalmos 2" [Supplementary Concept] OR "Nanophthalmos 3" [Supplementary Concept] )) OR "Microphthalmos"[Mesh]) OR "Hyperopia"[Mesh]) OR "Hyperopia, High" [Supplementary Concept]) OR "Glaucoma, Angle-Closure"[Mesh]) AND "Intraocular Pressure"[Mesh]) OR "Vision, Ocular"[Mesh]) OR "Refraction, Ocular"[Mesh]) OR "Anterior Chamber"[Mesh]) AND (((((((((((((((((((((((((cataract surgery[Title/Abstract]) OR (cataract extraction[Title/Abstract])) OR (laser peripheral iridotomy[Title/Abstract])) OR (microinvasive glaucoma surgery[Title/Abstract])) OR (procedure[Title/Abstract])) OR (laser[Title/Abstract])) OR (goniotomy[Title/Abstract]) OR (gonioplasty[Title/Abstract]) AND (adults[Title/Abstract])) AND (nanophthalmos[Title/Abstract])) OR (moderate hyperopia[Title/Abstract])) OR (severe hyperopia[Title/Abstract])) OR (hyperopia[Title/Abstract])) OR (microophthalmos[Title/Abstract])) OR (narrow angles[Title/Abstract])) OR (narrow angle glaucoma[Title/Abstract])) OR (angle closure[Title/Abstract])) OR (plateau iris[Title/Abstract])) OR (phacomorphic glaucoma[Title/Abstract])) OR (malignant glaucoma[Title/Abstract]) OR (aqueous misdirection[Title/Abstract]) AND (intraocular pressure[Title/Abstract])) OR (vision[Title/Abstract])) OR (degree of hyperopia[Title/Abstract])) OR (anterior chamber depth[Title/Abstract])) OR (grade of angle[Title/Abstract])) OR (effectiveness[Title/Abstract])) OR (refraction[Title/Abstract])) OR (medications[Title/Abstract])) |
| **Scopus** | TITLE-ABS-KEY ( ( nanophthalmos OR hyperopia OR microphthalmos OR (angle AND closure) OR (plateau AND iris) OR (phacomorphic AND glaucoma) OR (lens-induced AND glaucoma) OR (narrow AND angle AND glaucoma) OR (narrow AND angles) OR (malignant AND glaucoma) OR (aqueous AND misdirection)) AND adults AND ( (cataract AND surgery) OR lpi OR (laser AND peripheral AND iridotomy) OR laser OR surgery OR migs OR (minimally-invasive AND glaucoma) AND surgery OR (micro-invasive AND glaucoma AND surgery) OR procedure OR goniotomy OR (Laser AND goniotomy) OR gonioplasty) AND ((intraocular AND pressure) OR iop OR vision OR (visual AND acuity) OR (degree AND of AND hyperopia) OR (grade AND of AND angle) OR effectiveness OR (number AND of AND medications) OR refraction OR (anterior AND chamber AND depth))) AND PUBYEAR > 2009 AND PUBYEAR < 2025 |
| **Ovid MEDLINE** | ((nanophthalmos or hyperopia or microphthalmos or (angle and closure) or (plateau and iris) or (phacomorphic and glaucoma) or (lens-induced and glaucoma) or (narrow and angle and glaucoma) or (narrow and angles) or (malignant and glaucoma) or (aqueous and misdirection)) and adults and ((((cataract and surgery) or lpi or (laser and peripheral and iridotomy) or laser or surgery or migs or (minimally-invasive and glaucoma)) and surgery) or (micro-invasive and glaucoma and surgery) or procedure or goniotomy or (Laser and goniotomy) or gonioplasty) and ((intraocular and pressure) or iop or vision or (visual and acuity) or (degree and of and hyperopia) or (grade and of and angle) or effectiveness or (number and of and medications) or refraction or (anterior and chamber and depth))) |
| **Embase via Ovid** | (nanophthalmos:ti,ab,kw OR hyperopia:ti,ab,kw OR microphthalmos:ti,ab,kw OR (angle:ti,ab,kw AND closure:ti,ab,kw) OR (plateau:ti,ab,kw AND iris:ti,ab,kw) OR (phacomorphic:ti,ab,kw AND glaucoma:ti,ab,kw) OR ('lens induced':ti,ab,kw AND glaucoma:ti,ab,kw) OR (narrow:ti,ab,kw AND angle:ti,ab,kw AND glaucoma:ti,ab,kw) OR (narrow:ti,ab,kw AND angles:ti,ab,kw) OR (malignant:ti,ab,kw AND glaucoma:ti,ab,kw) OR (aqueous:ti,ab,kw AND misdirection:ti,ab,kw)) AND adults:ti,ab,kw AND ((cataract:ti,ab,kw AND surgery:ti,ab,kw OR lpi:ti,ab,kw OR (laser:ti,ab,kw AND peripheral:ti,ab,kw AND iridotomy:ti,ab,kw) OR laser:ti,ab,kw OR surgery:ti,ab,kw OR migs:ti,ab,kw OR ('minimally invasive':ti,ab,kw AND glaucoma:ti,ab,kw)) AND surgery:ti,ab,kw OR ('micro invasive':ti,ab,kw AND glaucoma:ti,ab,kw AND surgery:ti,ab,kw) OR procedure:ti,ab,kw OR goniotomy:ti,ab,kw OR (laser:ti,ab,kw AND goniotomy:ti,ab,kw) OR gonioplasty:ti,ab,kw) AND (intraocular:ti,ab,kw AND pressure:ti,ab,kw OR iop:ti,ab,kw OR vision:ti,ab,kw OR (visual:ti,ab,kw AND acuity:ti,ab,kw) OR (degree:ti,ab,kw AND of:ti,ab,kw AND hyperopia:ti,ab,kw) OR (grade:ti,ab,kw AND of:ti,ab,kw AND angle:ti,ab,kw) OR effectiveness:ti,ab,kw OR (number:ti,ab,kw AND of:ti,ab,kw AND medications:ti,ab,kw) OR refraction:ti,ab,kw OR (anterior:ti,ab,kw AND chamber:ti,ab,kw AND depth:ti,ab,kw)) |

**Supplemental Table 2.** Characteristics and results of included studies.

| **First Author, Year** | **Underlying Conditions (No. of eyes in group)** | **Intervention Pursued (No. of eyes in group)** | **Outcome Measurements** | **Complications** |
| --- | --- | --- | --- | --- |
| Alfonso, 2019 | High hyperopia (105) | Phacoemulsification with IOL implantation (105) | IOP, CDVA, UDVA, ACD, Refraction (SE) | None |
| Chen, 2014 | High hyperopia (11) | Phacoemulsification with IOL implantation (11) | ACD | 1 case of severe posterior capsular fibrosis |
| Elwehidy, 2020 | PACG with cataract (59) | Phacoemulsification with viscosynechiolysis (30)  Phacoemulsification with trabeculectomy (29) | IOP, CDVA, ACD | Hyphema, flat bleb, filtering bleb, Descemet membrane detachment, IOP elevation, corneal edema, fibrin exudation, shallow anterior chamber |
| Fan, 2023 | Nanophthalmos with angle-closure glaucoma (44) | Phacoemulsification with anterior vitrectomy and sclerotomy (19)  Phacoemulsification with anterior vitrectomy (15)  Filtering surgery (10) | IOP, BCVA, ACD, Refraction (SE) | Phaco with anterior vitrectomy and sclerotomy: Corneal edema, severe anterior chamber reaction, shallow anterior chamber, fibrin membrane, suprachoroidal hemorrhage, uveal effusion.  Phaco with anterior vitrectomy: Corneal edema, severe anterior chamber reaction, hyphema, shallow anterior chamber, vitreous hemorrhage, suprachoroidal hemorrhage, uveal effusion, retinal detachment.  Filtering surgery: Corneal edema, corneal endothelial decompensation, shallow anterior chamber, fibrin membrane, uveal effusion, retinal detachment, drainage tube obstruction, malignant glaucoma. |
| Jung, 2012 | Nanophthalmos (17)  Relative anterior microphthalmos (29)  Normal control (54) | Phacoemulsification with IOL implantation (100) | IOP, BCVA, ACD | Early postoperative corneal edema, anterior segment inflammation. |
| Kannan, 2024 | Iridofundal coloboma associated with microphthalmos and brown/mature cataract (15) | Phacoemulsification with IOL implantation (15) | IOP, CDVA | 1 patient underwent re-couching; no other major complications were noted. |
| Lin, 2023 | Nanophthalmos (26)  Relative anterior microphthalmos (12) | Phacoemulsification with IOL implantation (38) | ACD | None |
| Mohebbi, 2017 | Nanophthalmos or high hyperopia >40 D (18) | Refractive lens exchange with both IOLs in bag (7)  Refractive lens exchange with 1 IOL in bag, 1 in sulcus (11) | UDVA, CDVA, ACD, Refraction (SE) | Reduced vision due to IOL capture, iris fold, secondary angle closure, and IOP elevation, PCO. No patients with ILO. |
| Mustafa, 2019 | Microphthalmos  AL < 21 mm (45) | Phacoemulsification with IOL implantation (45) | ACD | None |
| Nicula, 2020 | Hyperopia (128) | Refractive lens exchange with both IOLs in bag (128) | CDVA, ACD, Refraction (SE) | Photic phenomena (glares, haloes), PCO, unsatisfactory vision quality (likely neuroadaptation). |
| Rajendrababu, 2021 | Nanophthalmos and cataract (144) | Small incision cataract surgery with sclerostomy, SICS without sclerostomy, phacoemulsification with sclerostomy, phacoemulsification without sclerostomy (114)  Trabeculectomy, trabeculectomy + sclerostomy, trabeculectomy + IOL + sclerostomy (11) | IOP | Cataract surgery complications (intra- and post-op): Whole bag removal, uveal effusion syndrome, fibrin membrane, corneal edema, vitreous disturbance, aqueous misdirection, capsular phimosis, exudative retinal detachment, posterior capsule rupture, zonular dialysis, shallow anterior chamber, iris corneal touch.  Glaucoma surgery complications: Intraoperative supra-choroidal hemorrhage, post-operative shallow anterior chamber, aqueous misdirection, choroidal detachment, severe fibrin reaction, tube reposition. |
| Rajendrababu, 2017 | Nanophthalmos and cataract (60) | Laser peripheral iridotomy 2 weeks prior to cataract surgery with concomitant prophylactic sclerostomy (29)  Laser peripheral iridotomy 2 weeks prior to cataract surgery alone (31) | IOP, BCVA, ACD, Refraction (SE) | Sclerostomy:  Descemet membrane stripping, fibrin membrane, retinal detachment.  Control: Iridodialysis, posterior capsular tear, rhexis extension, zonular dialysis, persistent corneal edema, anterior capsular phimosis, aqueous misdirection, choroidal effusion, fibrin membrane. |
| Seki, 2012 | Nanophthalmos (11) | Phacoemulsification with IOL implantation (11) | IOP, BCVA, ACD, Refraction (SE) | Difficult AC formation, iris herniation, partial zonular rupture, posterior synechiae, peripheral anterior synechiae, capsular block, aqueous misdirection, reocclusion of PI, iris bombe, IOP elevation, shallow AC. |
| Singh, 2015 | Nanophthalmos (21) | Phacoemulsification with IOL implantation (21) | IOP, UDVA, CDVA, ACD, Refraction (SE) | Malignant glaucoma, vitreous hemorrhage, retinal detachment, positive dysphotopsia, early IOP spike, corneal edema. |
| Steijns, 2013 | Nanophthalmos (43) | Phacoemulsification with IOL implantation (43) | BCVA, Refraction (SE) | Capsular defect without loss of lens fragments, capsular defect with loss of lens fragments, angle-closure glaucoma, retained lens material, severe iritis, corneal decompensation, anterior capsular phimosis, uveal effusion without serous retinal detachment, uveal effusion with serous retinal detachment, CME. |
| Ucar, 2022 | Nanophthalmos (61) | Phacoemulsification with  piggyback implantation of a 1-piece IOL  along with a 3-piece IOL into bag (31)  Phacoemulsification with piggyback implantation  of two 1-piece IOLs  into bag (30) | UDVA, ACD | Transient IOP elevation, posterior capsule opacification. |
| Wei, 2022 | Complex nanophthalmos (45) | Limited pars plana vitrectomy + phacoemulsification with IOL implantation + posterior capsulotomy (45) | IOP, BCVA, ACD | Localized choroidal detachment. |
| Xia, 2016 | Nanophthalmos or microphthalmos (40) | 1-site phacoemulsification combined with trabeculectomy (26)  2-site phacoemulsification combined with trabeculectomy (14) | IOP, BCVA, ACD | Switch to extracapsular cataract extraction because of crowded and shallow anterior chamber, clear corneal incision was sutured because of leakage, and corneal stroma edema. |
| Yosar, 2021 | Microphthalmos (71) | Phacoemulsification with IOL implantation (71) | IOP, CDVA, ACD, Refraction (SE) | Iris prolapse, corneal endothelial, Descemet membrane trauma, transient severe corneal edema, and cystoid macular edema (CME). |
| Zhang, 2017 | ACG secondary to nanophthalmos (21) | 23-G pars plana vitrectomy (PPV) combined with lensectomy (PPL) (21) | IOP, BCVA, ACD | Uveal effusion, iris bleeding that spontaneously stopped, iatrogenic retinal hole. |
| Zheng, 2017 | Cataract of simple microphthalmos with an AL <18 mm and without associated congenital ocular abnormalities (11)  Cataract of complex microphthalmos with an AL <18 mm and associated congenital ocular pathology (8)  Cataract of relative anterior microphthalmos with a CD <8 mm (11) | Phacoemulsification with IOL implantation (30) | IOP, BCVA, ACD, Refraction (SE) | Early corneal edema, glaucoma, and posterior capsular opacification. Post-operative glaucoma. Severe complications included suprachoroidal hemorrhage, endothelial dysfunction, and retinal detachment. |

**Supplemental Figure 1.** Leave-one-out sensitivity analysis for RE outcomes in **A)** nanophthalmic eyes at medium-term follow-up, **B)** microphthalmic eyes at medium-term follow-up, and **C)** high hyperopic eyes at medium-term follow-up.

**A**

**
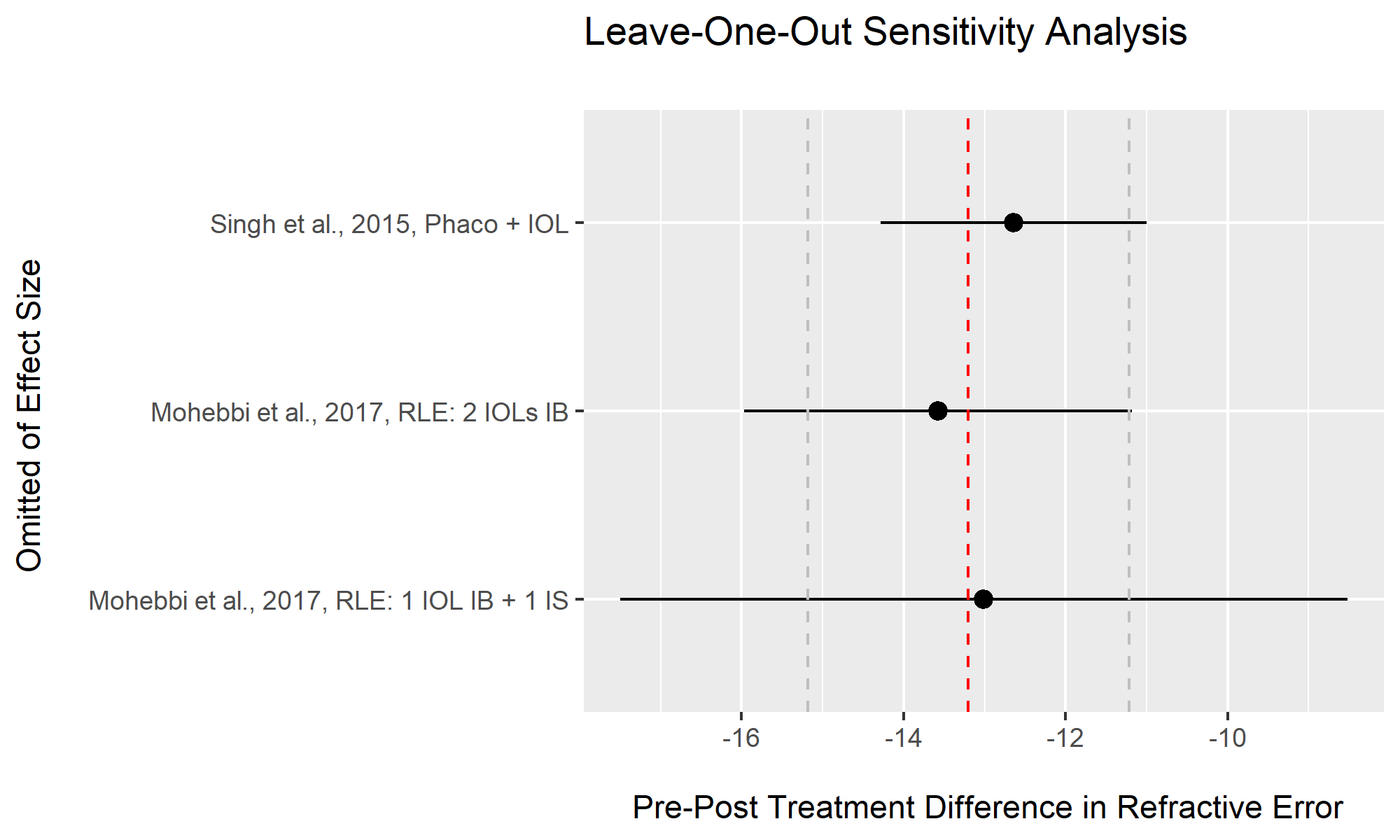
**

**B**

**
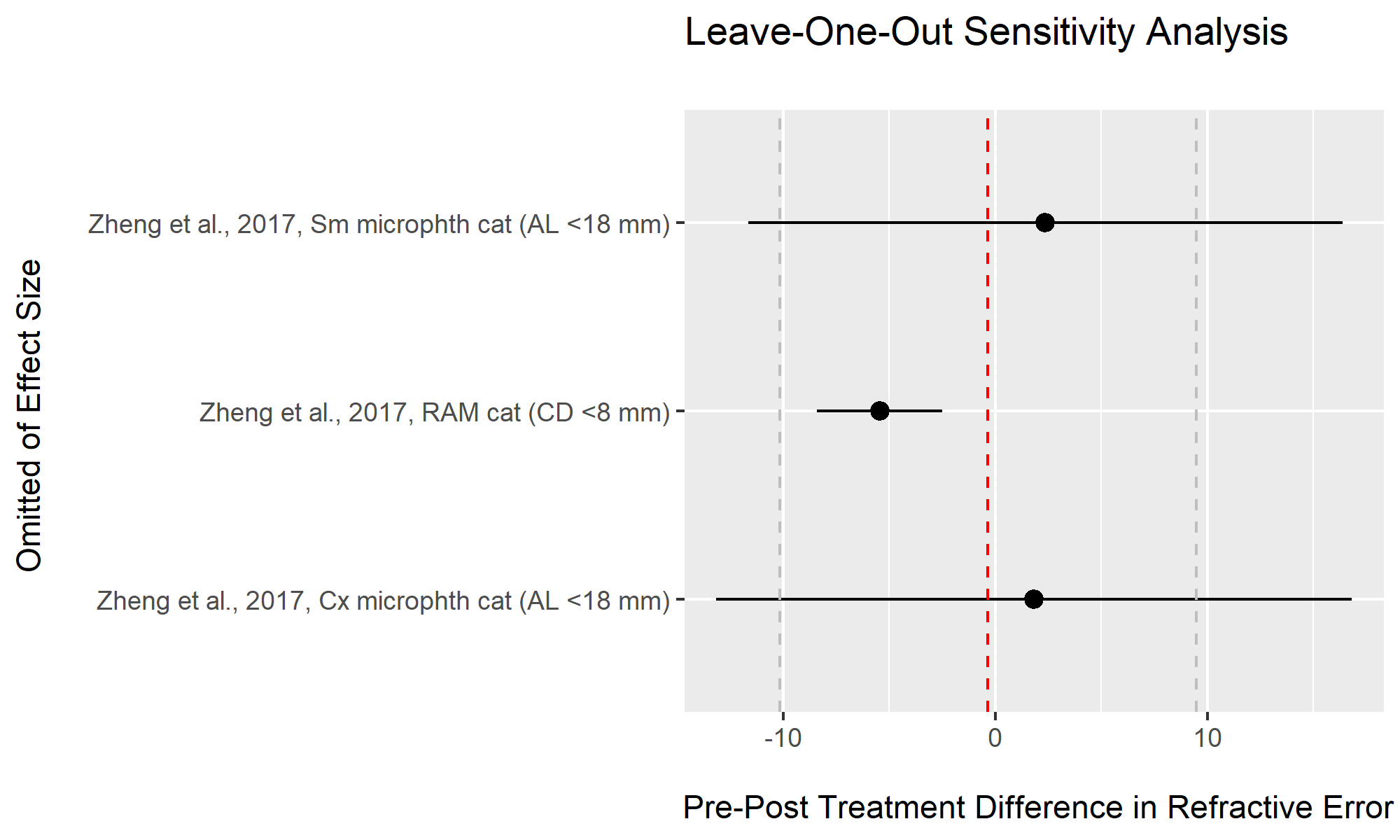
**

**C**

**
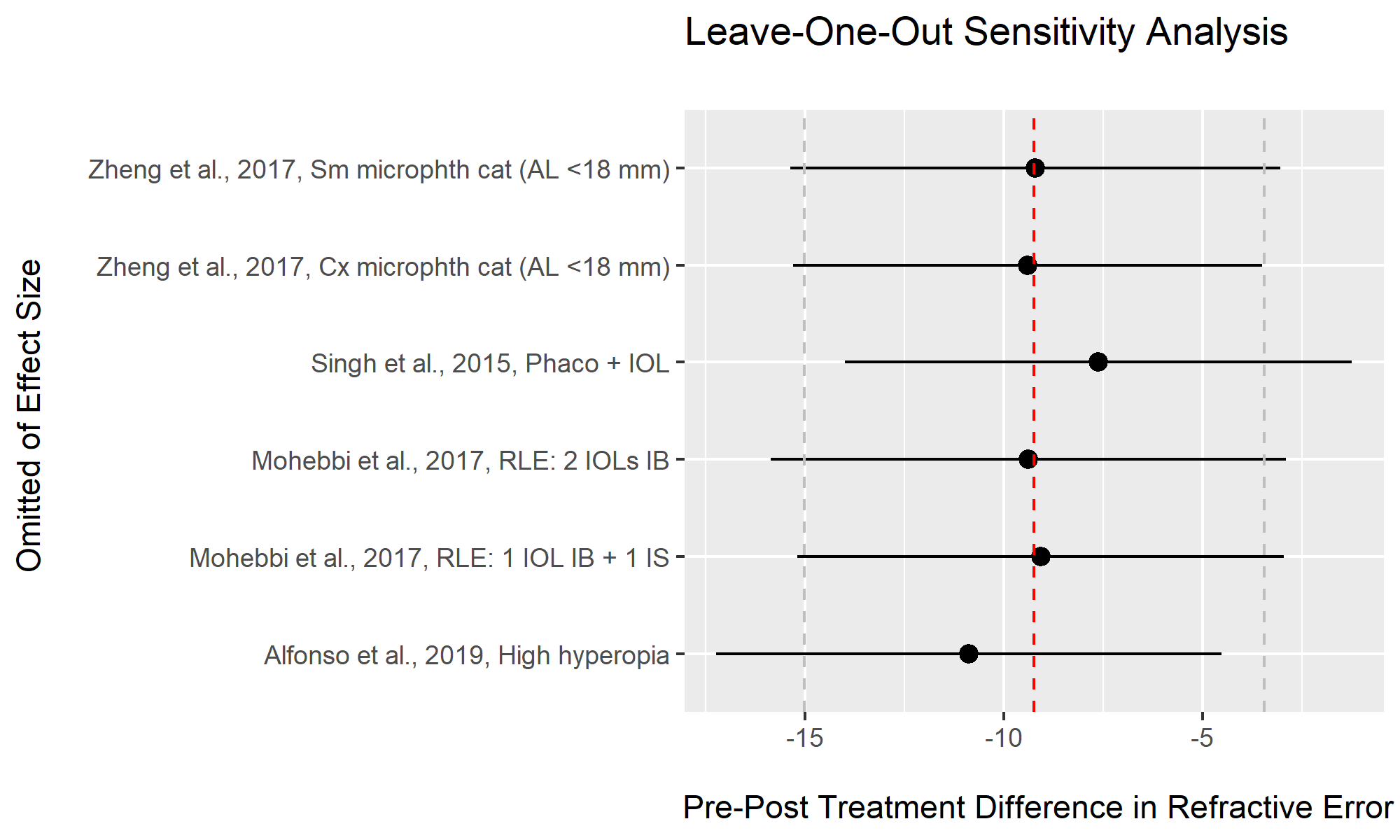
**

**Supplemental Figure 2.** Leave-one-out sensitivity analysis for VA outcomes in **A)** nanophthalmic eyes at short-term follow-up, **B)** nanophthalmic eyes at medium-term follow-up, and **C)** high hyperopic eyes at medium-term follow-up.


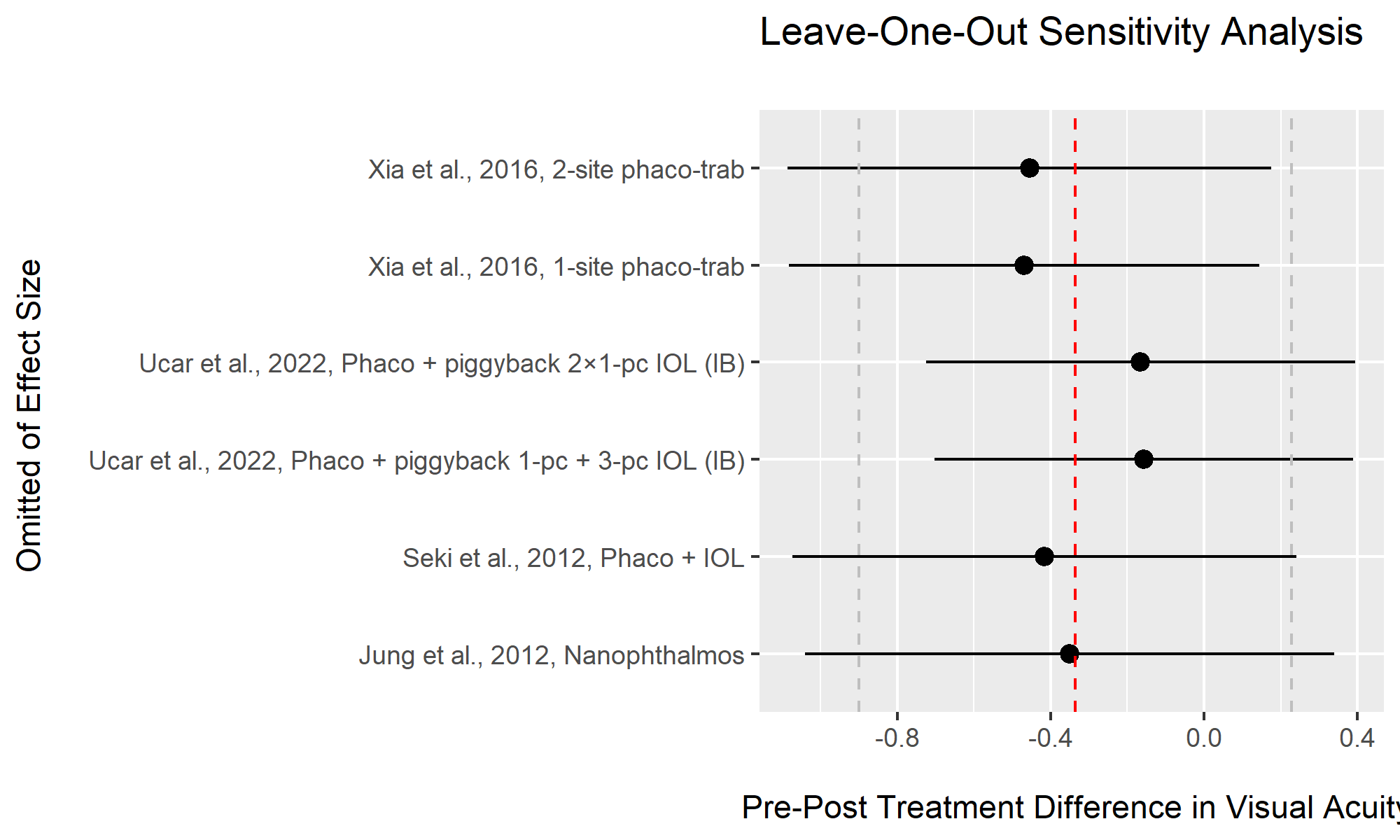


**A**

**
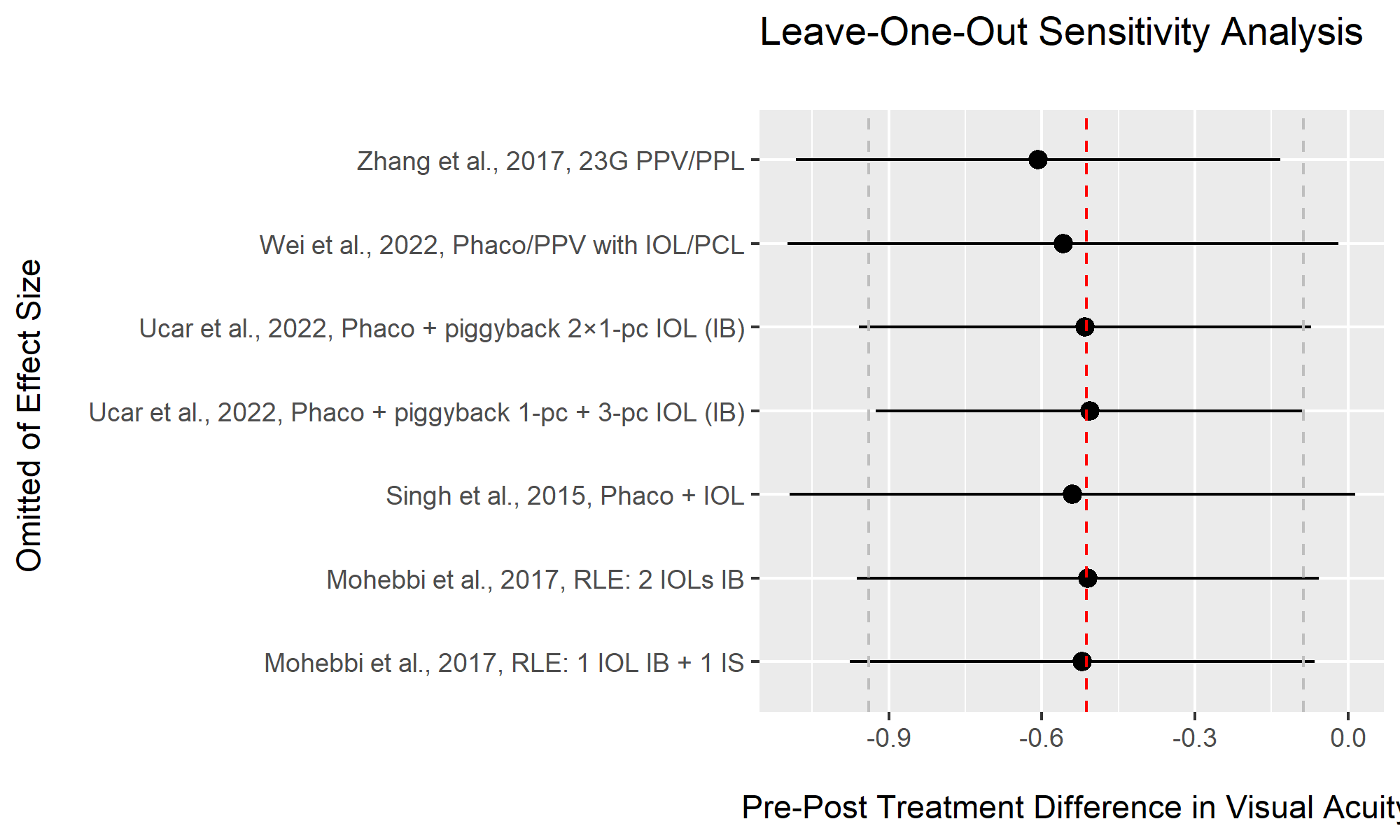
**

**B**

**
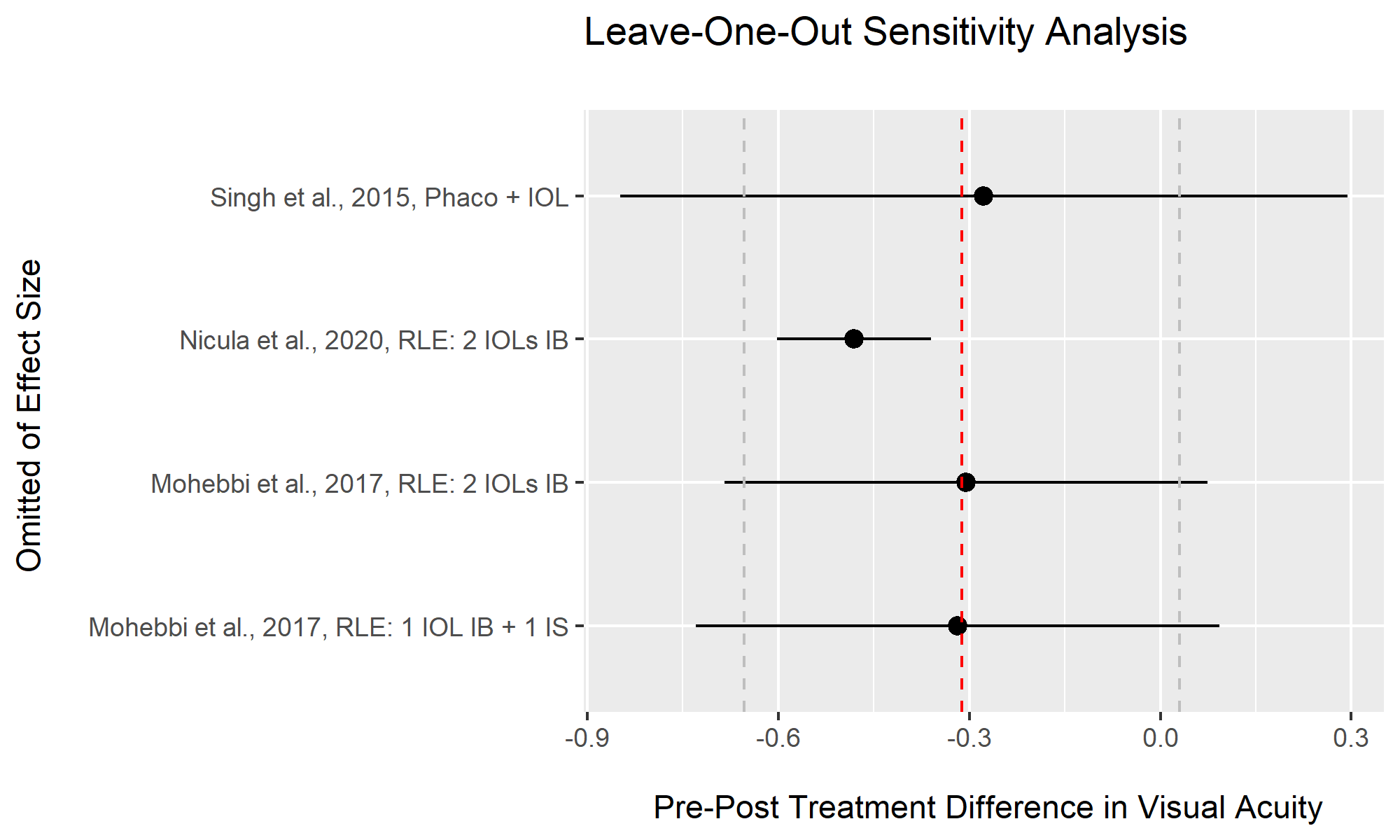
**

**C**

**Supplemental Figure 3.** Funnel plot and Egger’s regression test to assess publication bias for meta-analysis of short-term follow-up IOP outcomes in nanophthalmic eyes.


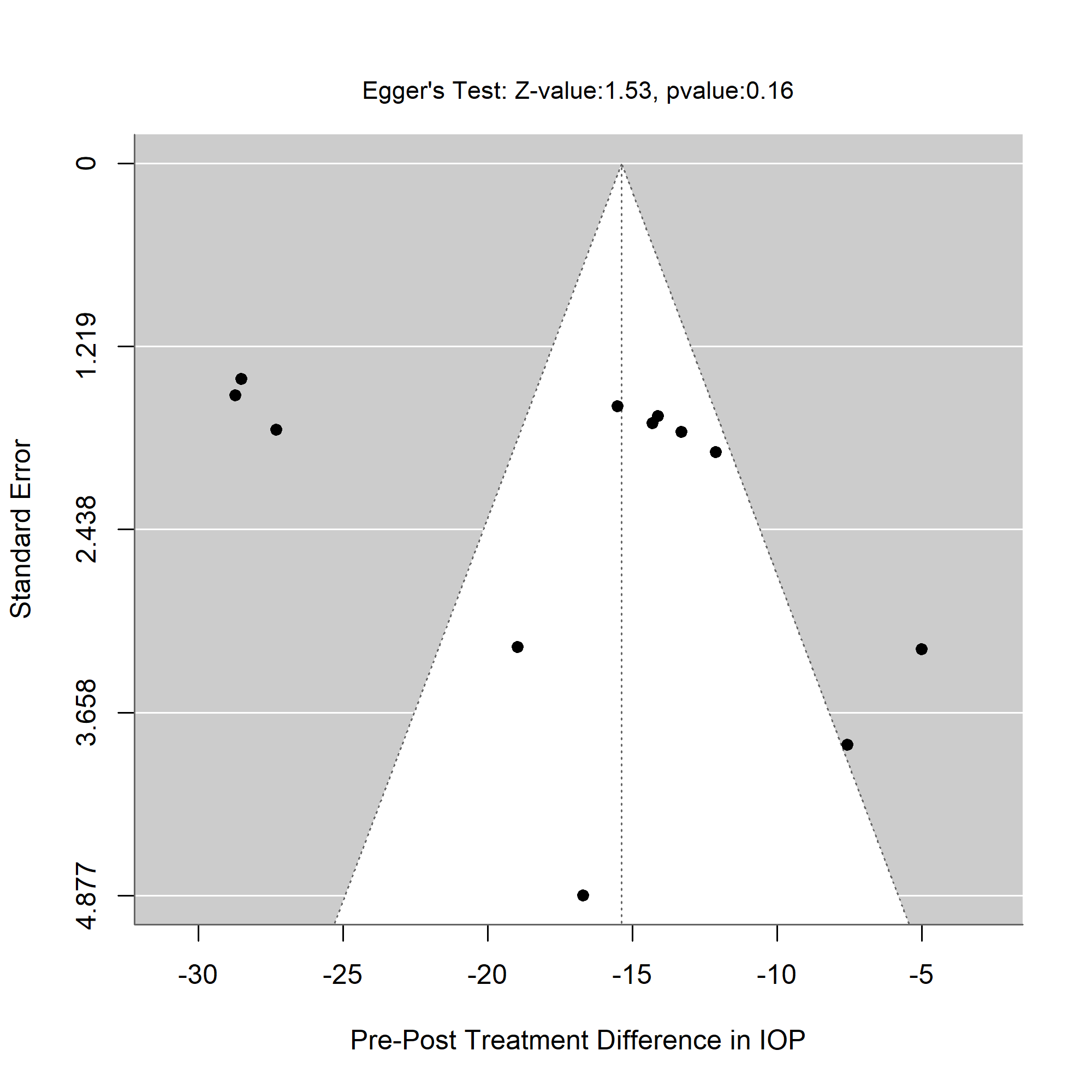


**Supplemental Figure 4.** Leave-one-out sensitivity analysis for IOP outcomes in **A)** nanophthalmic eyes at short-term follow-up, **B)** nanophthalmic eyes at medium-term follow-up, **C)** nanophthalmic eyes at long-term follow-up, and **D)** microphthalmic eyes at short-term follow-up.

**
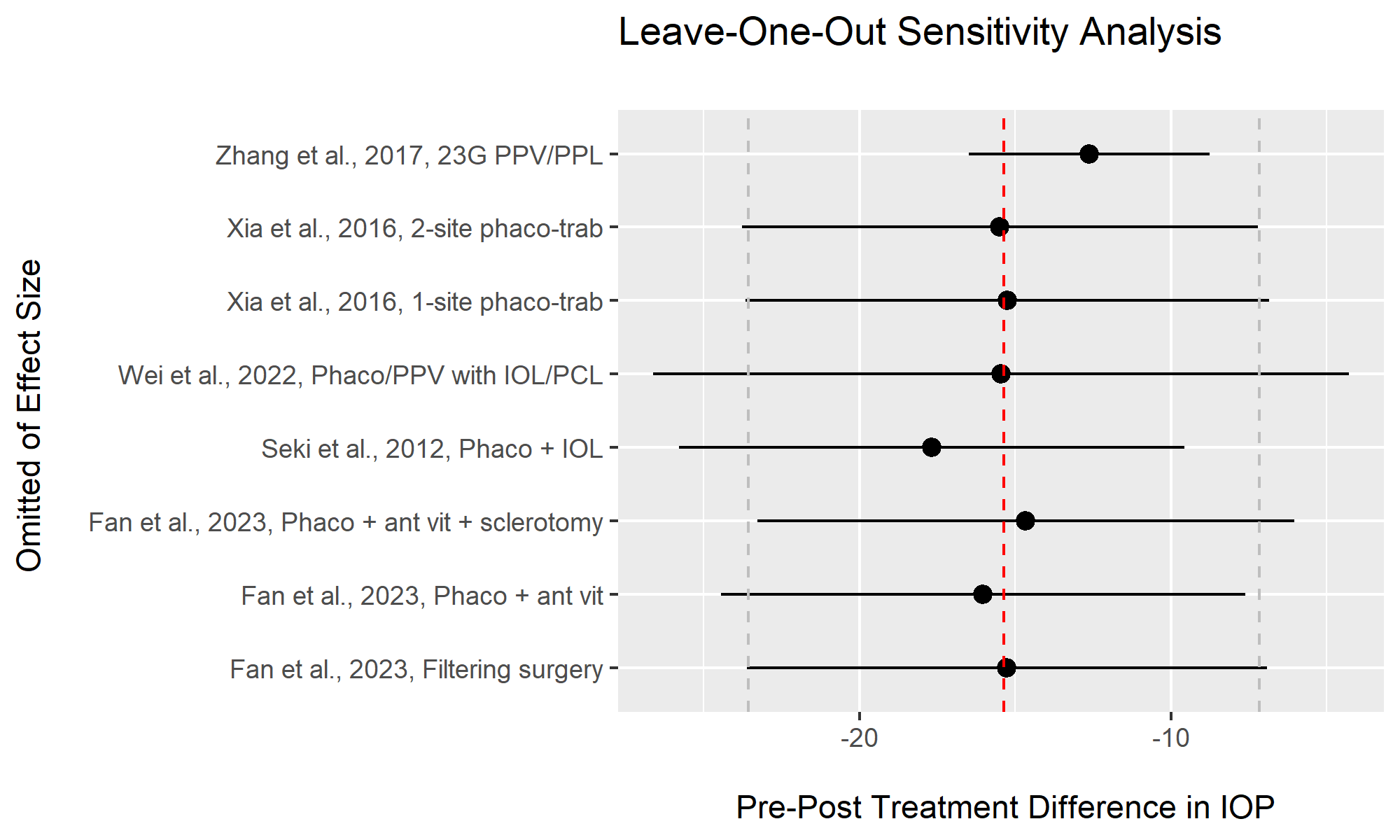

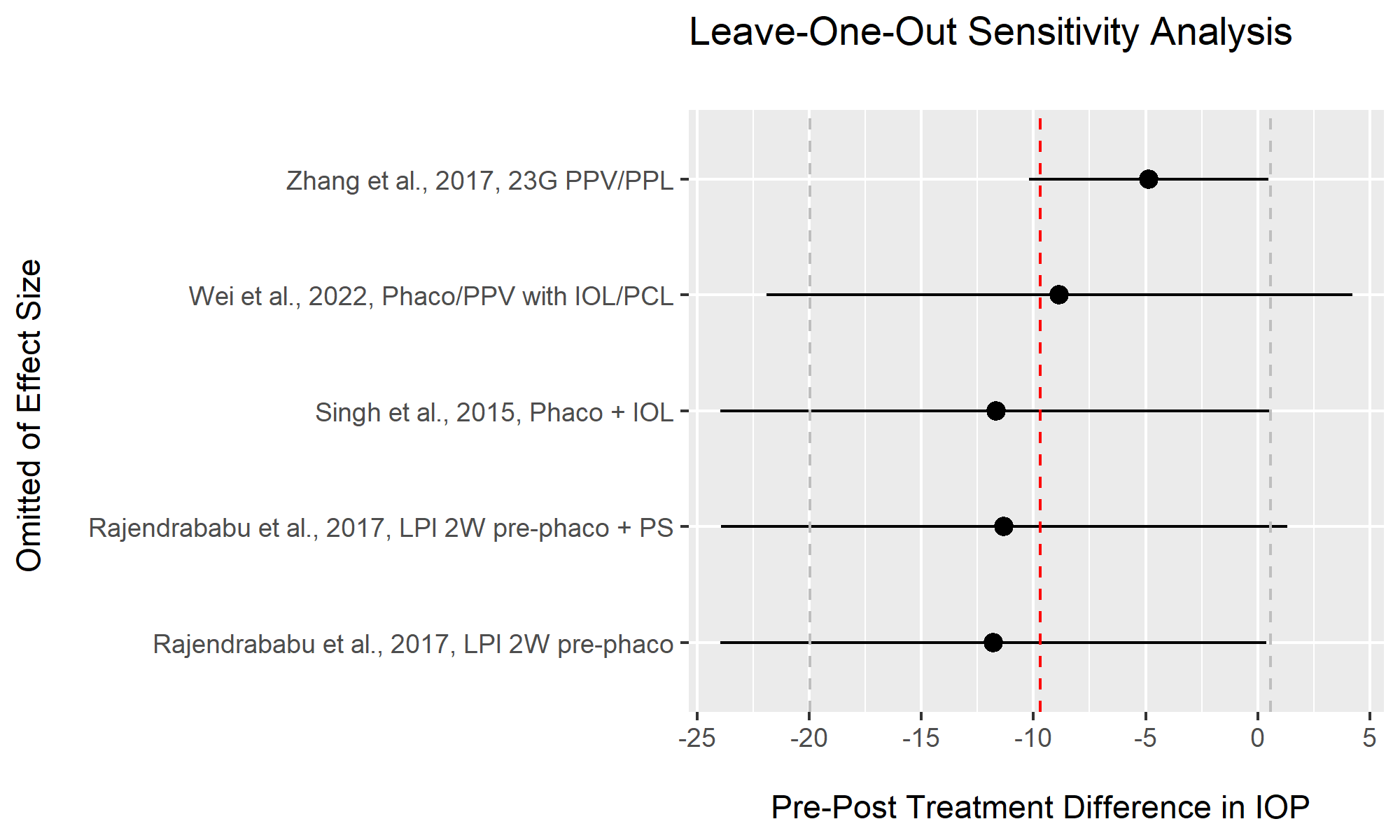
**

**B**

**A**

**
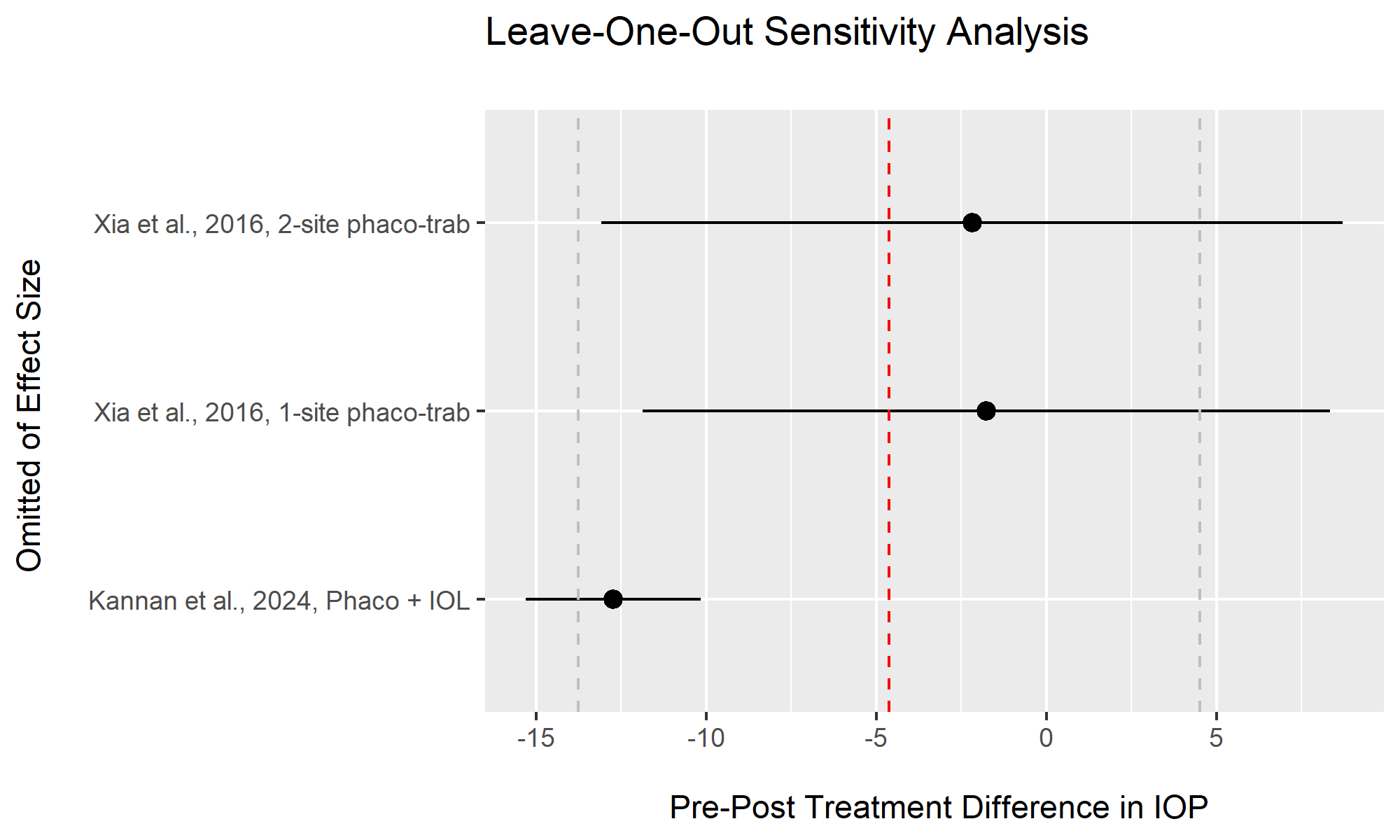
**

**D**

**C**

**
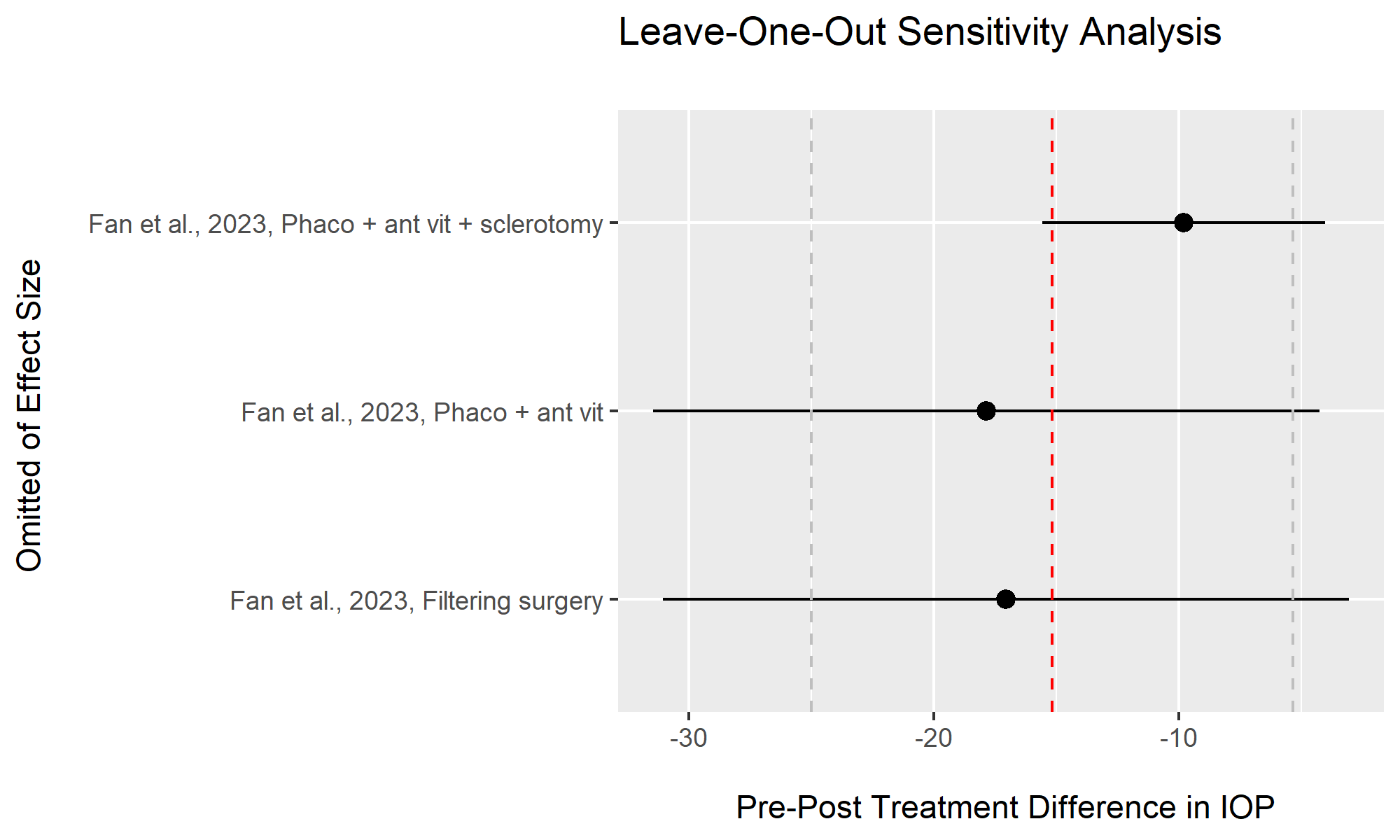
**

**Supplemental Figure 5.** Leave-one-out sensitivity analysis for ACD outcomes in **A)** nanophthalmic eyes at short-term follow-up, **B)** nanophthalmic eyes at medium-term follow-up, **C)** microphthalmic eyes at medium-term follow-up, and **D)** high hyperopic eyes at medium-term follow-up.

**
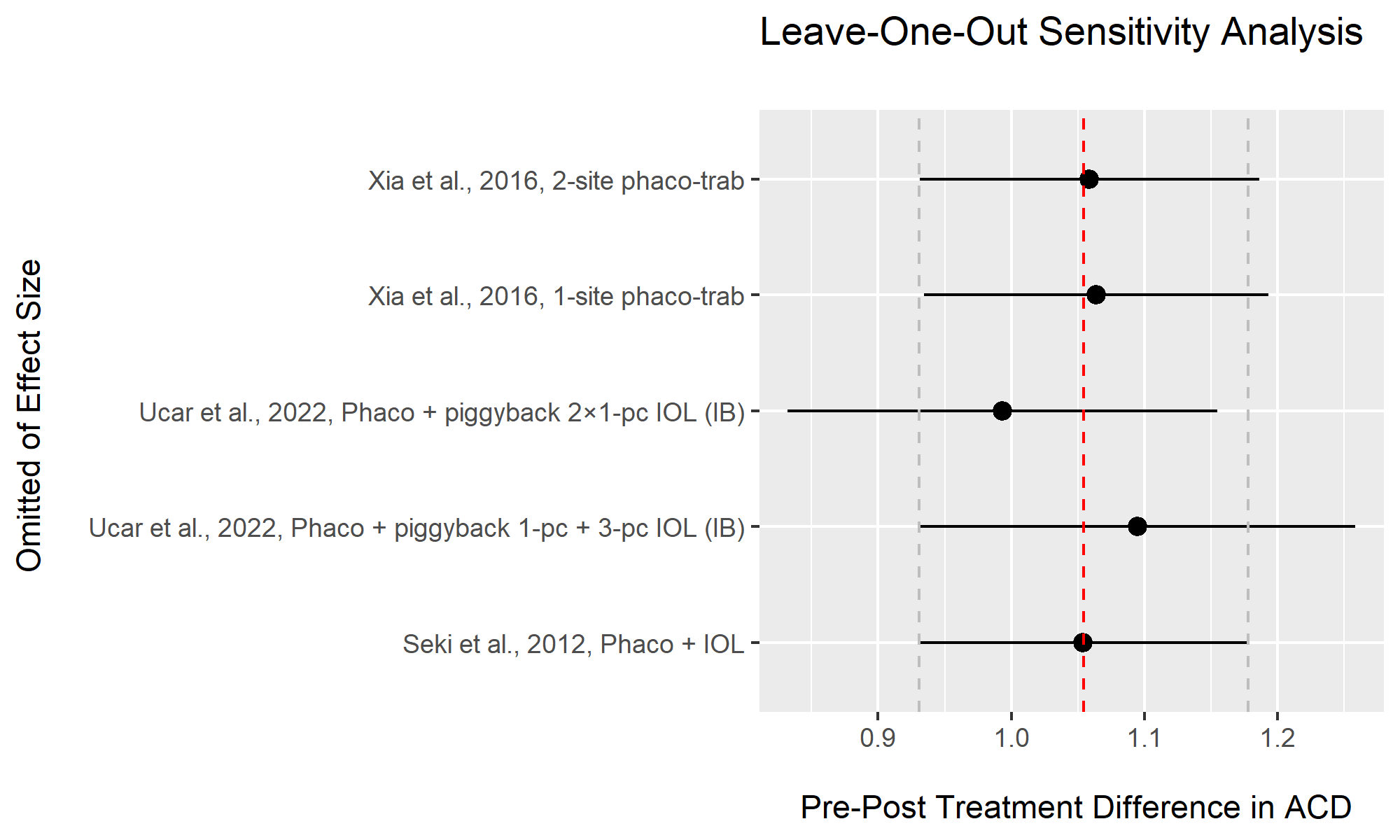

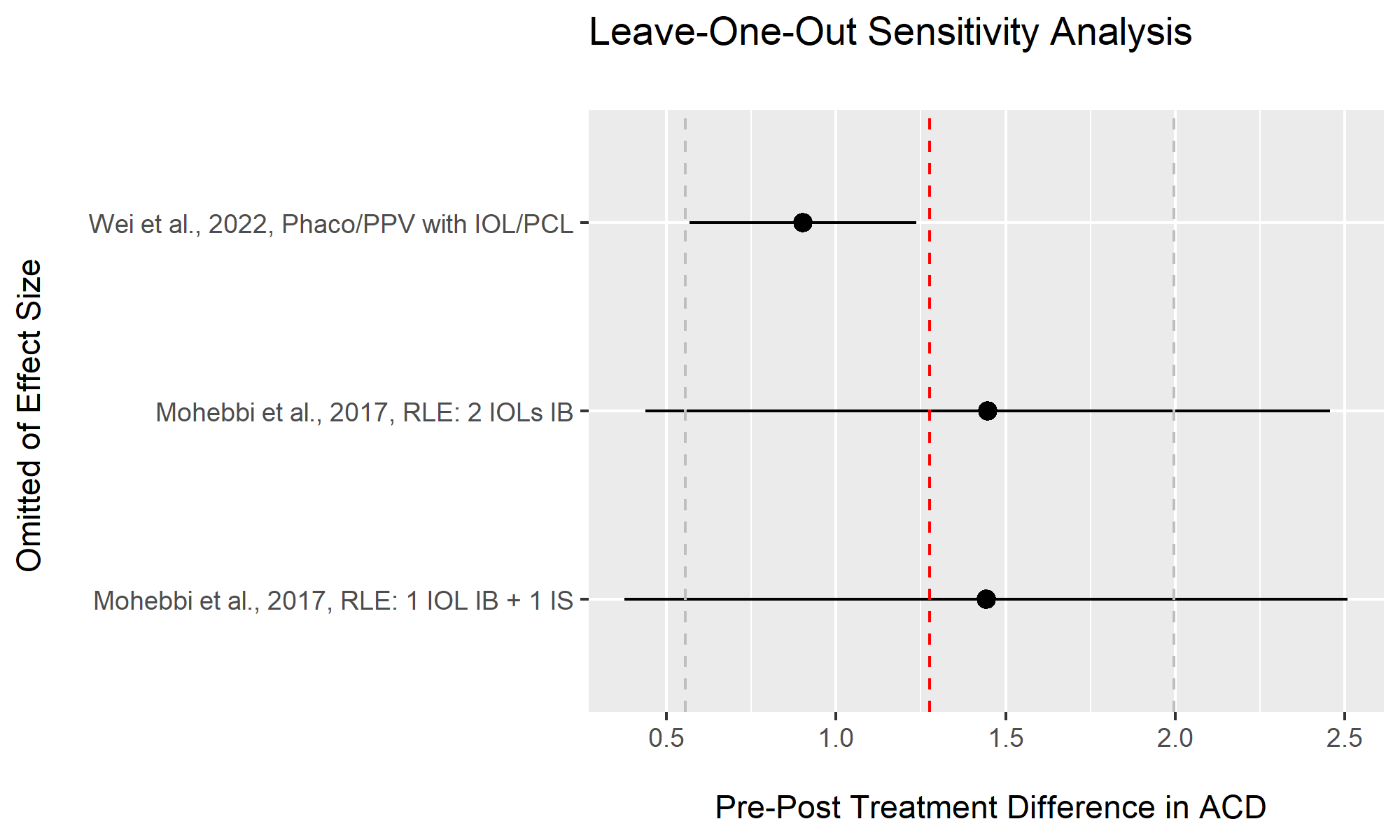
**

**A**

**B**

**C**

**D**

**
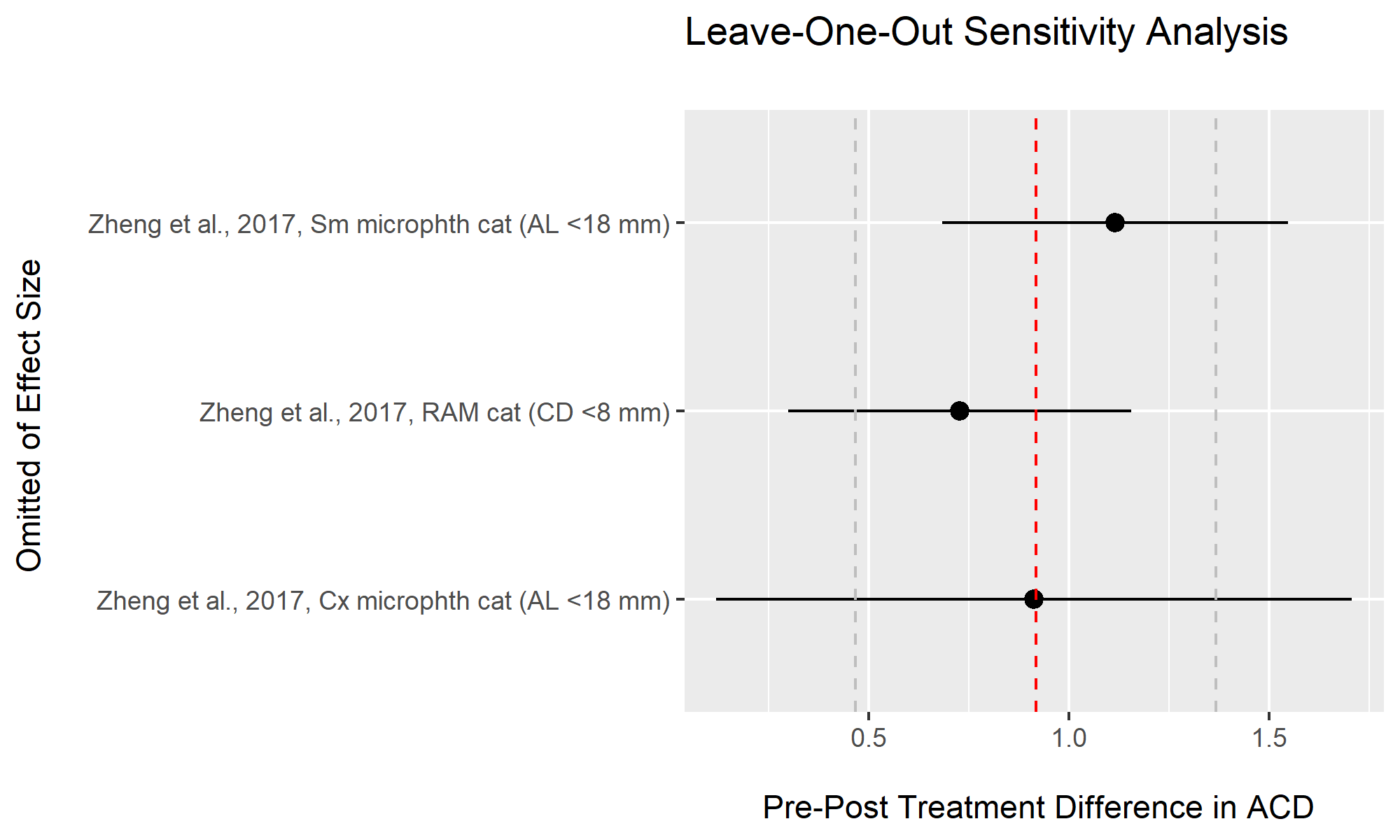

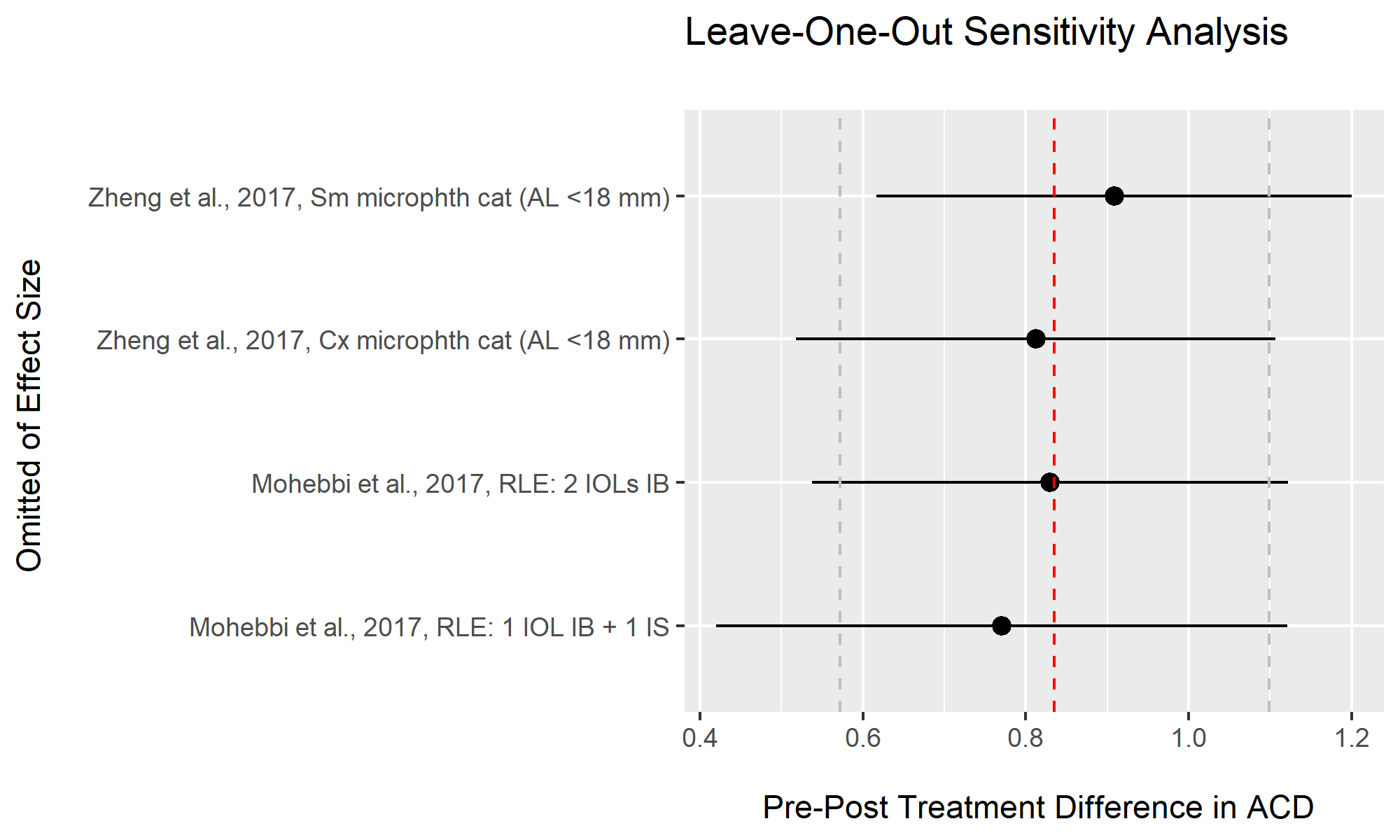
**

**Supplemental Figure 6.** Funnel plot and Egger’s regression test to assess publication bias for subgroup analysis of **A)** RE, **B)** VA, **C)** IOP, and **D)** ACD outcomes.


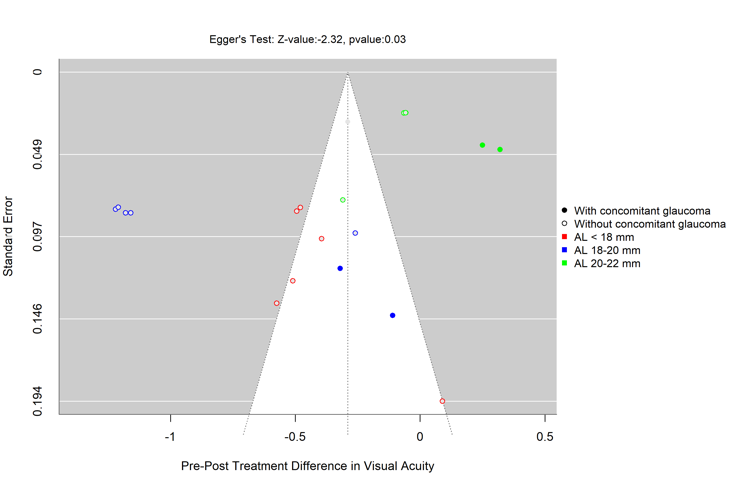

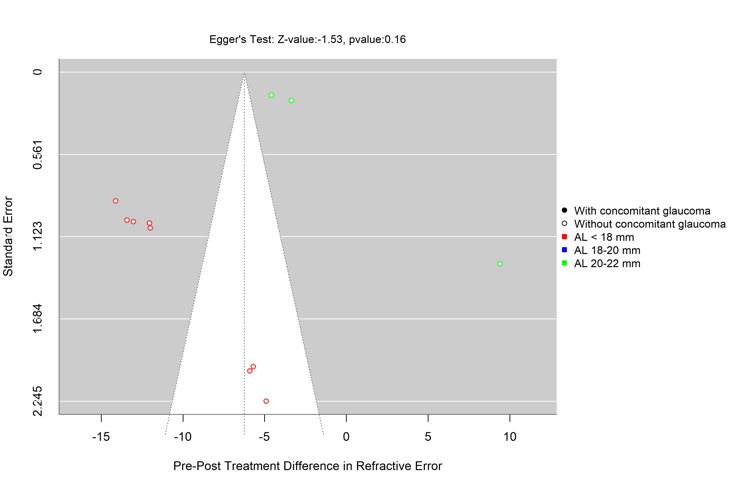

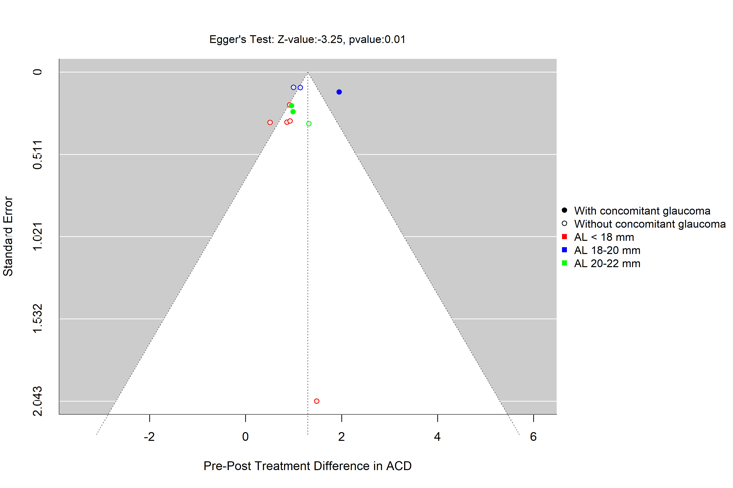

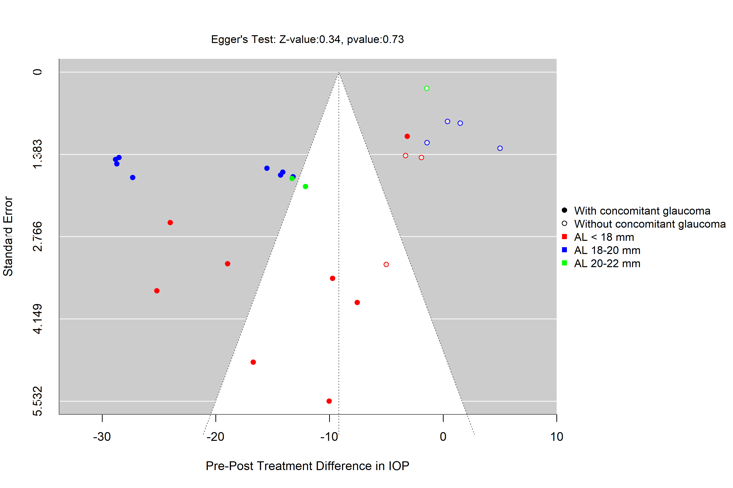


**D**

**B**

**C**

**A**
